# Citizens’ perceptions of the ethics of telepalliative care in the intensive care unit: a focus group study

**DOI:** 10.1186/s12916-026-05137-8

**Published:** 2026-08-18

**Authors:** Leen Krieckemans, Alice Cavolo, Michael Casaer, Chris Gastmans

**Affiliations:** 1 Centre for Biomedical Ethics and Law, Department of Public Health and Primary Care, KU Leuven Kapucijnenvoer 7 - box 7001, Leuven, 3000 Belgium; 2 Clinical Department and Laboratory of Intensive Care Medicine, Department of Cellular and Molecular Medicine, University Hospitals, KU Leuven Herestraat 49, Leuven, 3000 Belgium

**Keywords:** Palliative care, Intensive care medicine, Telemedicine, Bioethics, Ethical analysis, Citizen science, Qualitative research, Focus groups

## Abstract

**Background:**

Palliative care can be provided alongside curative or life-sustaining treatments and is not limited to the terminal phase, highlighting the need for integration across healthcare settings, including intensive care units (ICUs). Despite its benefits, ICU integration remains limited, leaving patient and family needs unmet. Telehealth, or telepalliative care, is increasingly investigated to improve ICU palliative care access, yet ethical implications remain unexplored. Early citizen consultation can reveal public concerns and facilitate implementation aligned with societal values. In contrast, failing to consider citizens’ perspectives may lead to resistance toward telepalliative care if perceived as conflicting with societal values. Therefore, this study investigates citizens’ perceptions of the ethical aspects of ICU telepalliative care.

**Methods:**

Constructivist grounded theory guided a qualitative study exploring citizens’ perspectives on ICU telepalliative care. Twelve focus groups (64 participants), including laypeople and healthcare professionals, were conducted in Flanders, Belgium. Data were analyzed iteratively using the Qualitative Analysis Guide of Leuven (QUAGOL). Reporting adhered to the Consolidated Criteria for Reporting Qualitative Research (COREQ) guidelines.

**Results:**

Participants questioned how telehealth could fit ICU palliative care, given conceptual tensions between telehealth, palliative care, and the ICU, and expressed concern that economic or technological motives might undermine ethical implementation. They recognized opportunities if guided by an ethical purpose with the human being at the center of care. Participants noted that telehealth both challenges and supports human contact: absence of physical presence, including touch, can limit relational aspects such as comfort, but virtual visits can help maintain contact when in-person interaction in the ICU is impossible. They also raised practical ethical concerns, including obtaining informed consent from unconscious patients and camera-related privacy risks, such as surveillance or disrupted intimacy, and proposed mitigations. Participants highlighted telehealth’s potential to improve equitable access to palliative care expertise in the ICU, while noting barriers such as limited digital literacy, language proficiency, and functional limitations.

**Conclusions:**

Citizens’ perspectives suggest ICU telepalliative care can be valuable under well-defined, human-centered conditions. Its ethical acceptability should be evaluated both for individual patients and for specific applications and continually revisited. Ethical implementation requires specifying use cases rather than treating “telehealth” abstractly.

## Background

Palliative care is increasingly recognized as an essential component of care, enhancing quality of life for patients with serious, life-limiting illness and their families^1^ by addressing physical, psychological, social, and spiritual needs [1]. It is not limited to the terminal phase and can be provided alongside curative or life-sustaining treatments, emphasizing the need for integration across healthcare settings rather than confinement to specialized units [1, 2]. The need for such integration is particularly evident in intensive care units (ICUs), where life-threatening conditions are often accompanied by prognostic uncertainty, complex treatment decisions, and substantial psychological and social distress for patients and families [3]. In this context, palliative care can, for example, support decision-making, facilitate discussions about goals of care, assist in conveying difficult information, and provide psychosocial and spiritual support [4, 5]. Palliative care in the ICU may be delivered by the ICU team itself, by specialist palliative care teams, or through a combination of both approaches [6]. Despite its benefits, integration of palliative care in the ICU remains limited, leaving many patient and family needs still unmet in practice [7]. Furthermore, there is a paucity of robust evidence regarding the effectiveness and cost-effectiveness of palliative care interventions in the ICU [8].

Telehealth is an innovative approach increasingly being investigated to improve access to palliative care, also referred to as telepalliative care [9–14]. It is currently also being explored to support the delivery of palliative care in ICU settings [15]. Telehealth is formally defined as:

…electronic information and telecommunication technologies that support long-distance clinical health care, patient and professional health-related education, public health and health administration. They are intended to allow monitoring and/or delivery of healthcare to patients at locations remote from where the healthcare professional is located [16].

Since the definition does not specify a single tool, telehealth can involve a variety of technologies, including videoconferencing, telemonitoring of vital signs, digital messaging platforms, and mobile applications. In addition to consultations between healthcare professionals, telehealth may also facilitate interactions between healthcare professionals and patients or their families, as well as between patients and family members. The potential diversity of technologies and interactions means that telepalliative care in the ICU can take many forms. For example, videoconferencing could allow ICU clinicians to consult with remote palliative care specialists on complex symptom management or goals-of-care decisions. Palliative care professionals may conduct virtual check-ins with ICU patients, supported by telemonitoring data, to assess symptoms and provide guidance or psychosocial support [17, 18]. Telehealth could also enable ICU clinicians to communicate with family members who cannot be physically present, using video or dedicated messaging platforms, and to facilitate interactions between patients and their families to maintain emotional connection [17, 18]. Despite its potential, robust evidence on the accessibility, and effectiveness of telepalliative care in the ICU remains scarce, with a notable lack of randomized controlled trials [8].

Telepalliative care in the ICU may be promising, but the introduction of telehealth as a new way of delivering palliative care may raise ethical questions that warrant careful examination. A recent review identified a broad range of ethical considerations in telepalliative care, including concerns related to all four major bioethical principles—respect for autonomy, beneficence, non-maleficence, and justice—as well as care-ethical concerns, such as implications for the patient–clinician relationship and the delivery of holistic care [19]. Similar results were reported in a recent review on the effectiveness and challenges of telepalliative care, albeit not in the ICU [20], where telepalliative care raised concerns in terms of accessibility, privacy, and acceptability. However, to date, no studies have examined the ethical issues that may arise specifically when telepalliative care is implemented in the ICU. This gap is important, as ethical issues are likely to depend not just on the specific technology in question, but also on the particular care context in which this technology is used [21]. Ethical analysis is also needed to ensure that telehealth innovations align with societal values and do not inadvertently conflict with them. Health technologies are shaped by social and historical contexts and may, in turn, reshape clinical practices and expectations [22]. Early involvement of citizens in implementation is therefore valuable, as it may help anticipate public concerns and support the introduction of telehealth in ways that are responsive to societal expectations [22–24]. Citizens bring a unique and valuable perspective to the ethical analysis as they can help identifying overlooked ethical concerns and their moral intuitions can guide policy. Further, involving citizens will also endorse the value of fairness as it gives people a voice in decisions that affect them. In doing so, it will also ensure that the implementation of telepalliative care in the ICU will reflect public values and maintain social trust [23]. Citizens’ perspectives include both laypeople, whose lack of professional expertise can help surface ethical concerns that experts might overlook [23], and individuals with relevant experience, such as healthcare professionals. Building on these considerations, this study aims to investigate the ethical aspects of ICU telepalliative care from the perspective of citizens. Specifically, our research question is: What do citizens perceive as the ethical aspects of telepalliative care in the intensive care unit? We focus on citizens living in Flanders, a region of roughly 6.8 million people in Belgium, where digital technologies are widely embedded in everyday life and healthcare [25].

## Methods

### Study design

This qualitative study employed a constructivist grounded theory design, in which findings are co-constructed by participants and researchers within their respective contexts [26, 27]. In this approach, researchers’ reflexivity and interpretation of the data are central. Because constructivist grounded theory generates insights inductively from the data rather than from predefined frameworks, it was particularly suited to the underexplored topic of ethical issues in ICU telepalliative care and enabled an in-depth exploration of citizens’ perspectives [27, 28].

Grounded theory involves the simultaneous collection and analysis of data, with each process informing the other. Through constant comparison between emerging data and ongoing analysis, we developed insights inductively while valuing the researchers’ interpretive contribution. Regular reflexive discussions within the research team helped avoid purely deductive reasoning. In addition, applying abductive logic enabled us to explore possible explanations for how citizens perceive the ethics of telepalliative care in ICU settings [29]. Rather than starting with a fixed theoretical framework (deduction) or building theory solely from data (induction), abductive logic involves an iterative process in which interpretations are refined through a continuous dialogue between empirical findings and theoretical insights.

Data were collected via focus groups to facilitate discussion and gather multiple perspectives efficiently. They are particularly valuable when little is known about a topic because they help identify key themes, language, and issues. Moreover, the interaction between participants stimulates discussion and can produce ideas that individuals may not think of alone [30]. The study protocol and abstract were registered on the Open Science Framework (OSF) (osf.io/f7xnh). Reporting adhered to the Consolidated Criteria for Reporting Qualitative Research (COREQ) guidelines [31].

### Participant recruitment and selection

Only participants who met the following eligibility criteria were included: (1) Belgian citizens with residence in Flanders, (2) at least 18 years old, (3) ability to speak Dutch, (4) ability to engage with video materials and participate in focus group discussions, (5) capacity to provide informed consent, and (6) willingness to participate.

A purposive sampling strategy was employed to ensure a diversity of perspectives, considering factors such as age, gender, educational level, cultural background, experience with palliative care, intensive care or telehealth (as a professional, relative, or patient), and familiarity with digital technologies. In addition, theoretical sampling was applied: participants were intentionally selected based on emerging insights from ongoing data analysis to further explore developing themes or gaps. For example, analysis of early focus groups indicated that individuals with limited language proficiency—which may be more common among cultural minorities—could face additional challenges using telehealth, partly due to reduced nonverbal communication. To explore this further, participants from these groups were deliberately recruited.

Recruitment was conducted through two complementary approaches. First, we collaborated with organizations, including socio-cultural groups, citizen organizations, and hospitals. These organizations received an information brochure and flyer via email. Depending on the organization’s preference, potential participants could either register directly with the researcher using the contact details provided, or submit their registration through the organization, which then forwarded the participant information to the research team. Second, recruitment was carried out through the research team’s professional network, with the study flyer distributed via social media platforms, institutional or publicly available email addresses, and in person at professional and community settings. Interested participants registered directly with the research team.

For study enrollment, potential participants received an information brochure containing detailed information about the study and the research team, including the researchers’ contact details, and a questionnaire assessing demographics, prior experience with palliative care, intensive care, telehealth, and self-rated digital competence. Eligible participants were then invited to attend a focus group based on their questionnaire responses.

### Data collection

Focus groups, comprising 2–8 participants, were conducted in Dutch between December 2024 and July 2025. As much as possible, focus groups were conformed in a way that all or most participants had some key characteristics in common (e.g. having or not having experience in palliative care, or religion). However, participants’ own preferences with regard to location and timings were also considered, meaning that pure homogeneity was not always possible. Sessions were led by the first author (LK), a 28 year old female PhD student in bioethics with a background in philosophy and psychology. She had prior qualitative research experience and practical experience in palliative care, complemented by ICU shadowing. She introduced herself to participants as a researcher in bioethics. An observer—a PhD student in bioethics with research experience in health technology and citizen involvement—was present in all sessions; in two cases, other PhD students in bioethics substituted. Focus groups were held either at locations provided by the participating organization or within the university. Each session began with brief participant introductions, including possible relevant experience, followed by a focusing exercise providing an introduction to palliative care, intensive care, and telehealth through videos, slides, and verbal explanations [32]. Palliative care was presented as focusing on quality of life and holistic care for patients with life-threatening illness and their relatives, compatible with curative treatment and not limited to the terminal phase. Examples of ICU palliative care that were provided included common clinical situations like care planning, family discussions, and coordination with life-support interventions. Telehealth was introduced broadly, in line with its definition, with examples including video consultations, remote monitoring, mobile applications and communication platforms. The PowerPoint presentation (in Dutch) used in the focusing exercise is provided in Additional file 1. The subsequent discussion was guided by a semi-structured topic guide (Additional file 2), informed by a prior systematic review [19] and refined iteratively during analysis. For example, a recurring theme that emerged was that telepalliative care in the ICU should focus on the human being. As this theme was initially vague, specific questions were added to the guide to clarify its concrete meaning. Sessions were audiotaped with participants’ consent, lasted 90–120 min (M = 103), and were transcribed verbatim. Data collection continued until theoretical saturation was reached, meaning that additional focus groups no longer contributed to the development or refinement of the emerging categories [28]. This occurred after ten focus groups. Two additional focus groups were conducted to confirm that the categories were sufficiently developed and that no further theoretical insights emerged. Field notes were taken by the observer during discussions, with additional reflections added by the moderator afterwards. Field notes were not included in the transcript and they were not analyzed. However, they were used to better contextualize the transcripts and quotes during the analysis.

### Data analysis

**Fig. 1:**
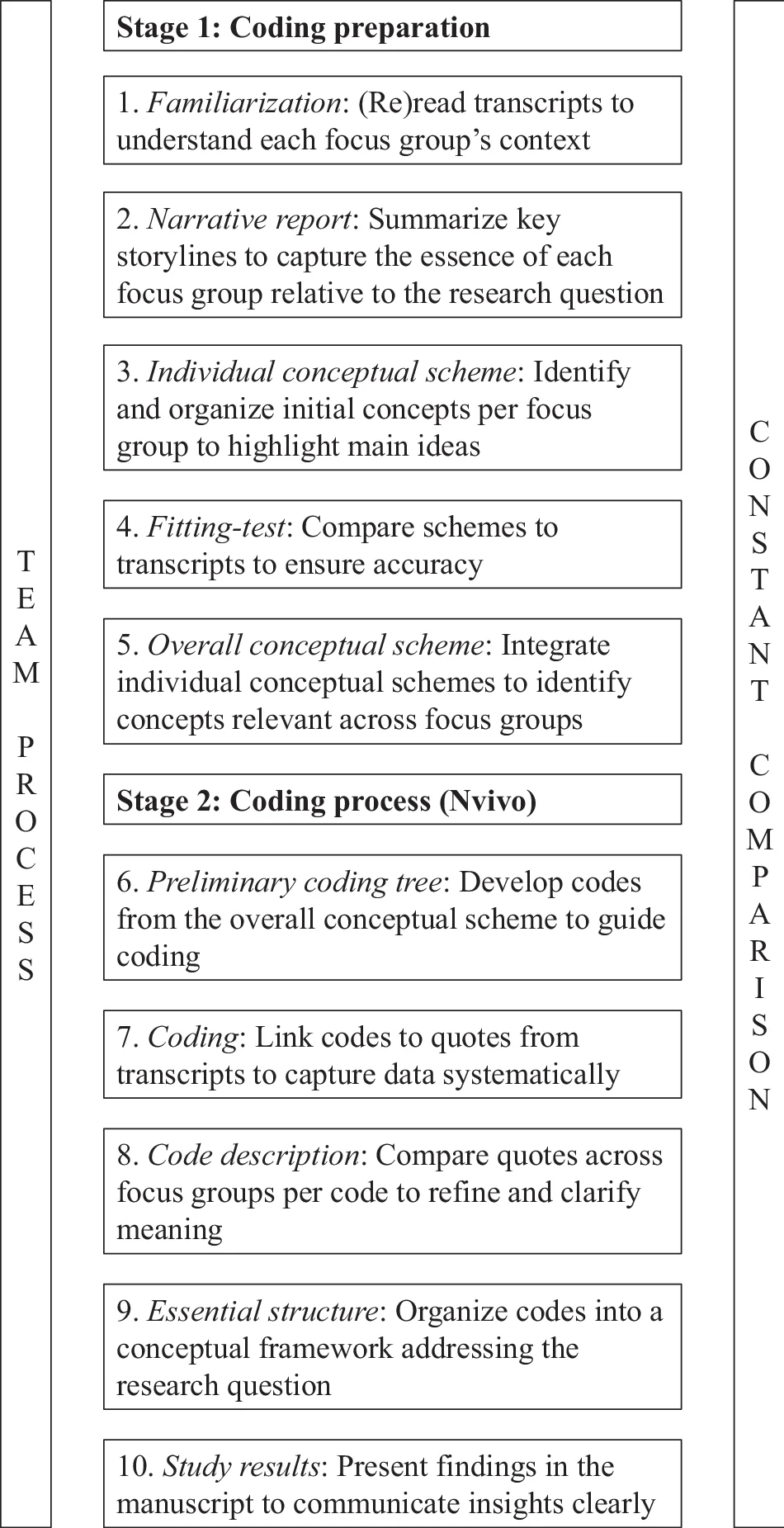
Qualitative analysis guide of Leuven (QUAGOL) workflow for data analysis. Modified from Dierckx de Casterlé et al. [33] and Vandemeulebroucke et al. [29]

Data analysis was conducted in Dutch. Only the quotes included in the manuscript were translated by the first author. Data analysis followed the Qualitative Analysis Guide of Leuven (QUAGOL) [33], a ten-stage approach using an iterative inductive–abductive process (Fig. 1). In line with grounded theory, data collection and analysis progressed concurrently.

The first phase focused on developing an initial conceptual understanding through the data, combining within-case analyses to retain contextual depth and across-case analyses to identify emerging patterns. An individual conceptual scheme was created for each focus group (see Additional file 3 for an example) to support this process. LK and CG conducted this phase independently, meeting regularly for in-depth discussions until consensus was reached. At the end of this phase, AC (a bioethicist) and MC (an intensivist) provided feedback on the emerging conceptual framework.

The second phase involved the actual coding process, supported by NVivo 15 [34]. The general conceptual scheme served as a basis to create a list of codes and relevant fragments from each transcript were coded in Nvivo 15. Each code was then analyzed using systematic within-case and across-case analysis. Concepts emerging from the analysis were organized and described in a comprehensive general framework, which served as the basis to report the results of our study. The conceptual framework developed in stage 1 was refined through repeated engagement with the transcripts to ensure it accurately represented the data. Primary coding was conducted by LK, with regular input from CG to ensure rigor. AC and MC provided feedback on the final analysis and on the general framework and reporting.

### Rigor

Rigor was ensured through multiple complementary strategies, including investigator triangulation, peer debriefing, expert opinion, and reflexivity [28, 35]. Investigator triangulation was applied during the conceptual phase of data analysis, with two researchers independently examining the data to minimize individual bias. The framework was then refined collaboratively during coding. Structural corroboration and coherence were supported through iterative analysis, continuously refining the emerging framework to ensure consistent and transparent interpretations. Peer debriefing involved discussions immediately after each focus group with an experienced observer, as well as follow-up team discussions shortly afterward. Expert opinion was sought from external experts in intensive care (MC) and bioethics (AC) to provide an independent check on the emerging framework. Throughout, reflexivity was maintained via memo writing and team discussions. An audit trail of memos and team discussions was kept by the first author

### Ethics

The study was approved by the Ethics Committee of KU Leuven (reference G-2024-7793-R2(MIN)) on 18 April 2024. Participants received written study information and the informed consent form in advance, which were also provided and discussed verbally at the start of the focus group. Written informed consent was obtained from all participants prior to participation. Participation was voluntary, and participants were free to withdraw at any time. All data were handled confidentially in accordance with the General Data Protection Regulation (GDPR), including pseudonymizing transcripts, restricting access to authorized team members, and secure storage.

A particular challenge was managing the sensitivity of the topic (palliative care and ICU) and potential emotional reactions. A continuous challenge throughout the focus groups was balancing a supportive and respectful response to participants’ emotions with the need to keep the discussion focused on the research topic. In practice, this required repeatedly negotiating when to allow space for personal stories and when to gently steer the discussion back to reflection on the topic. An independent observer attended all focus group sessions to monitor group dynamics and identify any signs of participant distress that might arise from the topics discussed. The observer was available to support the research team in addressing any ethical concerns should they occur. When situations of potential distress arose, they were managed, for example, by allowing time to offer emotional support or space to talk about a personal experience, or through peer support from other focus group members.

## Results

### Participant characteristics

A total of 64 participants took part in our study, distributed across 12 focus groups in four provinces in Flanders. Focus groups spanned from two to eight participants, with an average of five participants per group. One participant left a focus group about 15 min before it ended, without further explanation. Most participants were women (70.3%). The largest age group was 55–74 years (46.9%). Just over half had a university degree (51.6%), and participants reported a variety of religious or philosophical beliefs (Table 1).

**Table 1:** Participant demographics

Group number		1	2	3	4	5	6	7	8	9	10	11	12	Tot.	%
Number of participants		6	7	5	5	5	2	7	8	4	5	6	4	64	100.0
Gender	Woman	6	5	3	5	2	1	6	6	3	2	2	4	45	70.3
	Man	0	2	2	0	3	1	1	2	1	3	4	0	19	29.7
Age (years)	18–34	1	2	3	2	5	1	4	0	0	0	1	1	20	31.3
	35–54	2	1	1	0	0	0	1	0	3	2	0	3	13	20.3
	55–74	2	4	1	3	0	1	2	8	1	3	5	0	30	46.9
	75+	1	0	0	0	0	0	0	0	0	0	0	0	1	1.6
Level of education	Secondary education	1	2	0	0	0	0	0	4	0	0	0	0	7	10.9
	Higher education (non-university)	1	4	1	3	0	2	4	3	0	2	2	2	24	37.5
	University	4	1	4	2	5	0	3	1	4	3	4	2	33	51.6
Religious or philosophical belief	Christianity	4	1	3	3	0	0	2	7	3	0	5	0	28	43.8
	Islam	0	0	0	0	0	0	0	0	0	0	0	4	4	6.3
	Secular humanism or atheism	0	1	0	1	4	1	1	0	0	4	1	0	13	20.3
	No specific religion or belief	2	5	2	1	1	1	4	1	1	0	0	0	18	28.1
	Prefer not to say	0	0	0	0	0	0	0	0	0	1	0	0	1	1.6

Most participants had some experience with intensive care (57.8%) or palliative care (78.1%), either as a patient, relative, professional, or in multiple roles. Five groups consisted of expert citizens, meaning that all participants had relevant experience with palliative care or the ICU (either as a healthcare professional, as a relative, as a patient, or as a volunteer), and two groups had no healthcare professionals. This helped mitigating the risk of healthcare professionals influencing other participants’ opinions. Participants reported varying levels of digital competence and most (59.4%) reported no prior experience with telehealth (Table 2).

**Table 2:** Participant experience with intensive care, palliative care, and telehealth

Group number			1	2	3	4	5	6	7	8	9	10	11	12	Tot.	%
Number of participants			6	7	5	5	5	2	7	8	4	5	6	4	64	100.0
Experience with intensive care	No		4	2	1	2	5	0	3	3	0	1	4	2	27	42.2
	Yes		2	5	4	3	0	2	4	5	4	4	2	2	37	57.8
		*As a patient* ^a^	*0*	*1*	*2*	*0*	*0*	*0*	*0*	*1*	*0*	*2*	*0*	*0*	*6*	*9.4*
		*As a relative* ^a^	*2*	*4*	*2*	*2*	*0*	*0*	*1*	*3*	*1*	*1*	*1*	*2*	*19*	*29.7*
		*As a professional* ^a^	*0*	*1*	*0*	*2*	*0*	*2*	*4*	*2*	*4*	*1*	*2*	*0*	*18*	*28.1*
Experience with palliative care	No		1	1	0	0	4	0	0	2	0	2	2	2	14	21.9
	Yes		5	6	5	5	1	2	7	6	4	3	4	2	50	78.1
		*As a patient* ^a^	*0*	*1*	*0*	*0*	*0*	*0*	*0*	*0*	*0*	*0*	*0*	*0*	*1*	*1.6*
		*As a relative* ^a^	*3*	*3*	*4*	*4*	*1*	*1*	*2*	*1*	*2*	*1*	*3*	*2*	*27*	*42.2*
		*As a professional* ^a^	*4*	*2*	*1*	*4*	*0*	*2*	*6*	*2*	*4*	*2*	*2*	*0*	*29*	*45.3*
		*As a volunteer* ^a^	*1*	*1*	*0*	*0*	*0*	*0*	*0*	*3*	*0*	*1*	*0*	*0*	*6*	*9.3*
Self-rated digital competence	Very good		0	1	2	1	1	0	3	0	1	2	1	2	14	21.9
	Good		3	6	2	1	3	2	2	3	2	2	2	2	30	46.9
	Fair		1	0	0	2	1	0	1	5	0	1	3	0	14	21.9
	Poor		1	0	1	1	0	0	1	0	0	0	0	0	4	6.3
	Very Poor		1	0	0	0	0	0	0	0	1	0	0	0	2	3.1
Experience with telehealth	No		3	6	2	2	2	2	3	7	1	3	3	4	38	59.4
	Yes		2	1	3	3	3	0	3	0	3	2	3	0	23	35.9
		*As a patient* ^a^	*0*	*1*	*3*	*2*	*3*	*0*	*2*	*0*	*1*	*1*	*2*	*0*	*15*	*23.4*
		*As a professional* ^a^	*2*	*0*	*0*	*3*	*0*	*0*	*1*	*0*	*3*	*1*	*1*	*0*	*11*	*17.2*
	I don’t know what telehealth is		1	0	0	0	0	0	1	1	0	0	0	0	3	4.7

^a^Participants could select multiple subcategories; totals may exceed the number of participants in the category above

### Results overview

**Fig. 2:**
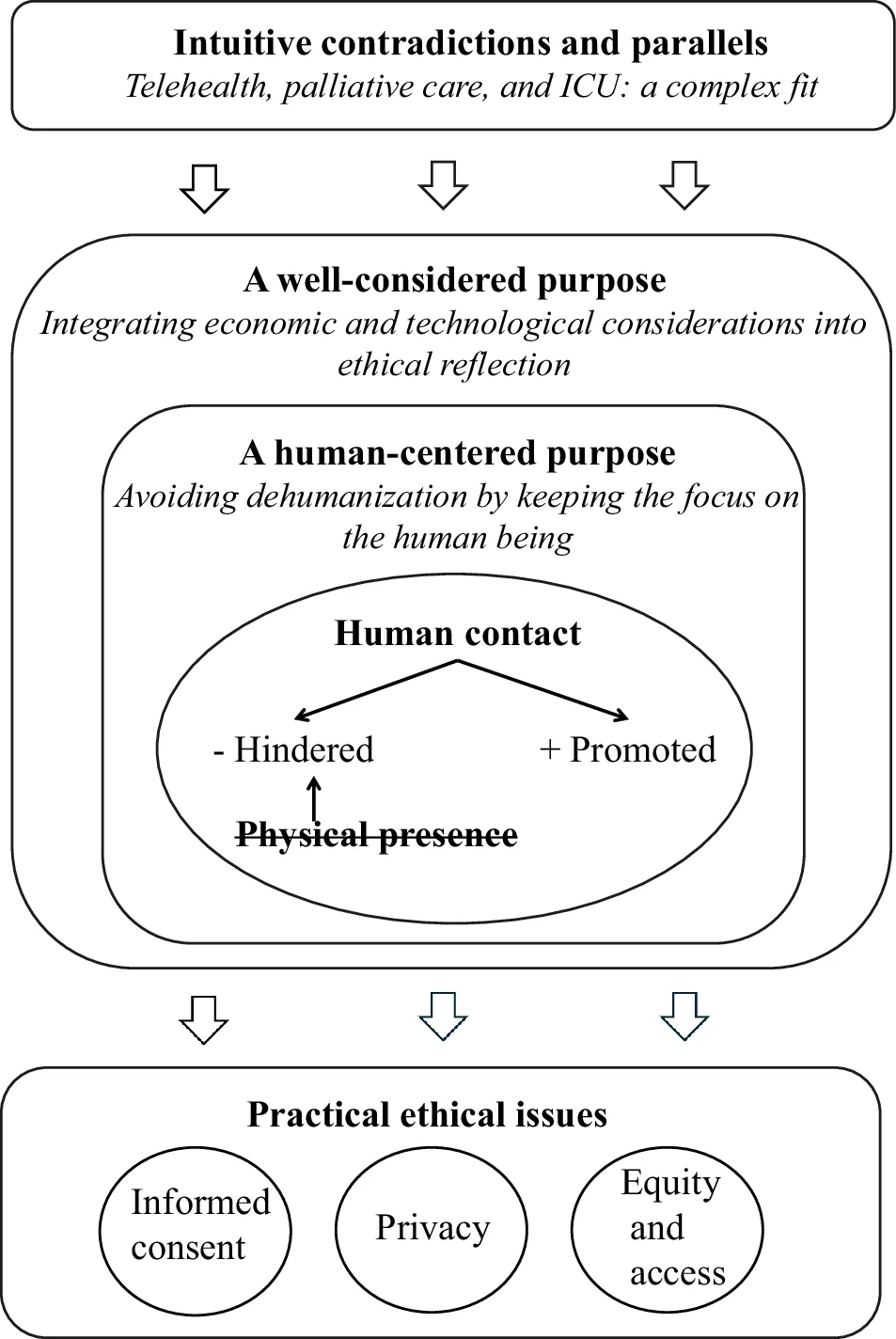
Conceptual framework of the ethical aspects of telepalliative care in the ICU derived from the data analysis

Participants initially found the topic challenging and did not intuitively see a role for telehealth in ICU palliative care, due to conceptual tensions between its constituting factors—telehealth, palliative care, and the ICU. After reflection, however, they recognized potential opportunities for its use, provided it is guided by a clear ethical purpose that keeps the human being at the center of care. To uphold this purpose, participants emphasized the importance of maintaining human contact, acknowledging that telepalliative care in the ICU presents both challenges and possibilities. They also highlighted practical ethical considerations, including informed consent, privacy, and equitable access. Figure 2 visually represents the conceptual framework of the ethical aspects of telepalliative care in the ICU derived from the data analysis.

### Theme 1: Intuitive contradictions and parallels

Throughout the focus group discussions, participants mainly drew on mental images of palliative care as focused on end-of-life situations, emphasizing holistic support for patients and their families. Intensive care was generally perceived as life-saving, focused on physical interventions within a highly technological environment. Telehealth was largely associated with video-based interactions, though participants also mentioned technologies such as remote monitoring and tablets, with interactions predominantly involving patients and families. When reflecting on telepalliative care in the ICU, participants intuitively perceived both contradictions and parallels between palliative care, intensive care, and telehealth. First, participants in nearly all focus groups (10/12) described intensive care and palliative care as differing in focus and setting. Participants characterized intensive care as life-saving, action-oriented, urgent, and primarily focused on the physical dimension, delivered in a technologically advanced environment. In contrast, palliative care was associated with acceptance of the end of life, a greater emphasis on being present rather than intervening, a slower pace, and attention to the person as a whole, often in a setting perceived as warmer and more homely. As one participant described:

FG2P2: “There’s also a contradiction between a curative focus — wanting to do everything to cure something — and palliative care, which is about being able to die well […]. For me, the ICU gives the impression of just going on and on. There are no limits, no boundaries, and it gives me the feeling: ‘Is it even allowed to die here? Or is it always just another tube, another line, another rescue?’ […] To me, these are two opposing worlds.”

Alongside these contrasts, participants highlighted areas of overlap between intensive care and palliative care (7/12 focus groups). They emphasized that palliative care should be an important part of ICU practice, supporting patients and families during critical decision points and at the end of life, which are common in the ICU. Participants also noted that ICU care already incorporates some elements of palliative care, including expertise around symptom control and increasing attention to quality of life. In addition, participants recognized that, in some cases, a short ICU stay can be integrated into a longer-term palliative trajectory, providing acute interventions when needed while maintaining a focus on long-term quality of life.

Second, many participants (8/12 focus groups) perceived a tension between palliative care and telehealth. They described palliative care as emphasizing close, attentive care, warmth, and taking time, whereas telehealth delivers care at a distance and creates a more technological environment with an emphasis on efficiency and speed. Participants were therefore hesitant about using telehealth in palliative care. At the same time, many participants (8/12 focus groups) were more accepting of telehealth in the ICU, noting that its highly technical, efficiency- and speed-focused approach parallels many characteristics of ICU care, making it especially effective for monitoring patients and supporting life-saving interventions. As one participant explained:

FG11P2: I don’t see how this [telehealth] could contribute to palliative care, to quality of life, or to psychological support. It all feels very technical, and the patient’s voice is nowhere to be found […] So for me, it has value in intensive care, but I don’t see its value in palliative care.

Not only were participants hesitant about using telehealth for palliative care; they were particularly reluctant to use telehealth for palliative care within an intensive care environment. The tension they already perceived between palliative care and intensive care was, in their view, further amplified by the introduction of telehealth. One participant illustrated this tension:

FG9P4: I also think we’re already in such a cold environment, full of alarms and monitoring […] and if the palliative care component is delivered the same way, then we’re taking an even further step away from humane intensive care.

### Theme 2: A well-considered ethical purpose

Although participants were initially hesitant about telehealth’s role in providing palliative care in the ICU, in most focus groups (9/12) its potential value was recognized if guided by a clearly articulated *ethical purpose*. They described this purpose as centering on the human being, safeguarding the core palliative care value of humanity. One participant noted:

FG11P2: We often return to the same question: where does the human dimension fit in all these new technologies? I don’t think palliative care can function without it. And whatever technology you introduce, if that isn’t considered, I doubt it will succeed.

Participants suggested that telepalliative care in the ICU should ideally deliver tangible benefits for patients, families, or staff—such as promoting well-being, enhancing connection, or supporting high-quality care—but, at a minimum, it must avoid dehumanization. Achieving this, participants mentioned, requires critical evaluation of these technologies. As one participant emphasized: “It’s important to check whether we’re really using this in the right way — not just going with the flow, but actually looking at where it can add value.” (FG11P3) Finally, participants noted potential tensions between an ethical purpose and competing purposes, particularly a *technological* and an *economic* one.

The first competing purpose identified by participants (9/12 focus groups) was *technological*, reflecting a perceived “technological imperative” characterized by a push toward ongoing innovation. As one participant explained: “This might sound a bit hopeless, but what keeps coming up for me is: we’re not going to be able to stop this - it feels so overpowering.” (FG11P6) They were concerned that such an imperative could lead to uncritical adoption of telepalliative care in the ICU. Participants did not oppose innovation per se but emphasized that technology should serve an ethical purpose rather than be an end in itself: “In my view, technology should only be used as a tool, and it should always stay subordinate.” (FG10P4)

The second competing purpose identified by participants (10/12 focus groups) was *economic*. The prospect that telepalliative care in the ICU could improve efficiency—such as by saving time or reducing staffing needs—and lower costs makes economic motivations particularly appealing amid ongoing staffing and resource pressures. As one participant cautioned:

FG7P4: What worries me is how far this will go, especially in times of shortages and scarcity, when people are looking for ways to save resources. While this may have some benefits, it also comes with many drawbacks. […] I just hope it doesn’t create a blind spot for what really matters.

As with technological innovation, participants did not reject economic considerations per se, but emphasized that these should remain secondary to an ethical purpose. They explained that efficiency gains should not undermine the human dimension of care and should ideally reinforce it. For patients, this implies reinvesting efficiency gains into direct care: “I think you can save a lot of time, but the question is: what do you do with that freed-up time? I believe it should be spent with the patients, since they should be the main focus.” (FG10P5) For care providers, efficiency gains should not translate into additional strain:

FG5P1: I think there are opportunities to share expertise and hold consultations via screens […], so they don’t have to travel as much and can see more patients in a day. But whether that’s actually better in this burnout-prone society, I don’t know—because the more of these consultations you sit through, the more it weighs on your mind.

Building on their concerns about technological and economic pressures, in all focus groups (12/12) participants emphasized that telepalliative care should not replace in-person palliative care for relational human-centered interactions. At the same time, participants acknowledged that telepalliative care in the ICU could serve as a “second best” option in well-defined circumstances: “Of course, there are emergencies—like during the COVID period—when this was a very welcome tool. But in everyday care, when it’s not necessary, I’m not sure what advantages it really offers.” (FG10P4) Beyond emergencies, participants identified telepalliative care as particularly suitable for informational or task-oriented interactions, such as exchanging medical information (e.g. bodily parameters) or coordinating practical aspects of care, such as arrangements with family members. They viewed this use not merely as acceptable, but as something that should be actively promoted. One participant emphasized: “To me it just seems logical that as long as it’s technical, we should be using all the tools available.” (FG5P3) When used for these non-emotional, task-focused interactions, participants thought that telepalliative care in the ICU can support and complement in-person palliative care:

FG2P2: I think the complementary aspect is really important. I don’t think we should exclude either one of them—they should complement each other to reinforce where we want to go. Hopefully, we can look at it in a balanced way […]

### Theme 3: Human contact as essential to realizing the ethical purpose

To maintain this ethical purpose while using telehealth, participants emphasized the importance of preserving high-quality human contact, a core component of ICU palliative care. They reported that, in general, telehealth can challenge this due to the absence of physical presence, but also described specific situations in which it may promote human contact. Finally, to support these benefits, they offered practical recommendations.

#### Sub-theme 3.1: Telehealth challenges essential human contact in ICU palliative care

One of the most discussed concerns (12/12 focus groups) was that telepalliative care in the ICU could disrupt human contact. Participants worried that, compared with in-person encounters, tele-interactions would be less rich, often describing them as less fulfilling, impersonal, cold, artificial, and distant. As one participant explained: “It all feels kind of robotic. A screen literally and figuratively creates distance.” (FG6P1) Participants emphasized that high-quality human contact is particularly important in contexts of heightened vulnerability, such as the ICU: “And just when you’re in intensive care, you’re at your most vulnerable as a human being, physically. And then it feels a bit wrong that there’s no one there who can just hold your hand once in a while.” (FG3P1) Against this background, participants identified several ICU palliative care situations where telehealth was considered less suitable. Delivering bad news, for example, was viewed as inappropriate:

FG7P6: I really believe those kinds of messages should, as much as possible, be given in person, at the bedside. These are moments that change people’s lives […] People remember them, where they heard it and who was there. And it’s not just about what’s said, but about the small details, about how it’s done. That’s not something you can convey over the phone, or even through a screen.

Advance care planning was likewise considered ill-suited for telehealth:

FG2P7: That’s about how you want to die and things like that, right? No. No.

FG2P6: It’s weird—we’re the generation that does everything by phone, but for something like that, I’d still find it really strange […]

FG2P5: I would never do something like that remotely.

Finally, participants generally did not consider telehealth adequate for supporting end-of-life farewells: “It’s just unbearable. You can’t put people through that. […] What kind of thing is that—having to say goodbye to their mom or their child through a screen? That’s just not something you should do.” (FG8P5)

#### Sub-theme 3.2: Physical presence as a condition for high-quality human contact

Participants highlighted that a key reason tele-interactions were perceived as falling short of full human contact was the absence of a physical dimension (10/12 focus groups). This physical dimension was seen as essential for relational aspects of care, both in patient-health care professional, patient-family and family-healthcare professional interactions. Even simple bodily presence of a family member or a healthcare professional was often described as a source of comfort. Participants noted that physical presence of relatives was especially important at the end of life:

FG5P5: I imagine people who are dying, people I’ve already lost, and it’s hard to picture them saying in those final days: ‘I want a video call.’ Very often, they just wanted someone to be there— even just reading a newspaper in the same room. Just someone present, not even to talk to and it wouldn’t even matter what kind of training they had […] I guess it’s really something very basic—[…] something fundamental—just presence in that moment.

Physical presence was also described as a sign of respect, signaling that the encounter matters – an expectation they saw as intrinsic to ICU palliative care. Reflecting on virtual family visits, one participant noted:

FG1P6: Yeah, I’d much rather not just do it online… Because if you do it like that, you’re not even making the effort to actually go there. […] In some cases, that’s just laziness. Not making any real effort to go see someone… […] It’s easier to just stay at home, turn that thing on: ‘So, how are you?’ Click, click, click, and done.

Extending this idea, participants emphasized that healthcare providers should be physically present whenever possible, viewing in-person presence as a fundamental sign of respect, particularly at the end of life.

Participants noted that the lack of physical presence in tele-interactions reduces key sensory cues—familiar smells, voices, and eye contact—that make encounters meaningful. One participant emphasized the importance of sensory experience in sensitive moments: “Putting them in front of a screen? That’s not how it should be! […] When it comes to moments like this: hold them, look them in the eyes! You need to be able to taste, touch, and experience people.” (FG8P5) Participants highlighted touch as crucial for providing comfort, whether from healthcare providers to patients, family to patients, or providers to relatives:

FG2P5: Imagine someone gets emotional […] I mean, I wouldn’t even know what to say if it’s digital. Like, “Don’t cry?” (awkward laughter)

FG2P2: Yeah, “Hold your screen, and I’ll hold mine!” (awkward laughter)

FG2P5: Exactly. Yeah… holding your screen… that’s really not it.

Physical presence and touch were also seen as essential for establishing trust in healthcare providers: “Yes, I would actually find it difficult if a doctor had never seen me in person… never looked at me, never touched me. How can you make any judgment about my health if you haven’t even touched me once?” (FG3P4)

Participants pointed out that the absence of physical presence not only reduces sensory cues but also limits nonverbal communication, which is crucial for interpreting emotions and responding empathically. Reflecting on the implications for healthcare professionals’ interactions with patients and relatives, one participant noted:

FG3P2: People don’t communicate only with words. You can still see the person, but you might miss some things. […] Some people might say that everything is fine, but then you notice them fidgeting. You just can’t see that on the screen […]—the body language, that subtle nuance.

Finally, participants described how the combined loss of sensory and nonverbal cues makes humor and silence harder to convey and interpret. One intensive care physician explained:

FG9P4: For me, humor is really important in intensive care, even when things are tough. But humor over a screen is really hard, because it can come across completely wrong. If you’re joking a bit and hold someone’s hand, they know, ‘He’s teasing me, but it’s okay.’ Behind a screen, I think that’s risky.

Similarly, a palliative care volunteer observed: “Sometimes silence speaks for itself. But over a screen, it’s like, ‘Hello? Hello? […] Can you still hear me?’—and that’s just not the same. I think a whole dimension is lost when you go digital.” (FG10P5)

#### Sub-theme 3.3: Promoting human contact in the ICU through telepalliative care

Although in-person encounters were considered ideal, in all focus groups (12/12) participants recognized that telepalliative care in the ICU provides a meaningful alternative when physical presence is not possible. Participants described tele-encounters as a valuable way to maintain connections between patients and families, particularly when relatives live abroad, face mobility limitations, or during restricted ICU visitation (e.g. COVID-19). In such situations, telehealth was considered acceptable even during vulnerable moments, including at the very end of life, to allow patients and family members to exchange final words. Participants (9/12 focus groups) highlighted that telepalliative care can also help to maintain family presence in the ICU, where visiting hours are often limited, by providing extra opportunities for contact. In their view, this flexibility could be especially valuable when commuting and other responsibilities, such as work or caregiving, make in-person visits overly burdensome for relatives. Finally, participants described additional contact opportunities as also potentially benefiting patients, who could be reassured by knowing they could always reach someone. One participant reflected:

FG9P4: Yes, but I was thinking… maybe we sometimes say too quickly, “Using those screens, it’s inhumane.” But I can imagine, if you’re there, sick but still somewhat capable, that it could be reassuring to just press a button and know your wife is on the other side […] to see her, even just for a minute […] I’ve never been in intensive care, thankfully […] But yes, I do think it’s important.

#### Sub-theme 3.4: Recommendations

Participants offered several recommendations to minimize disruptions to human contact in ICU telepalliative care. Almost all focus groups (11/12) emphasized that telepalliative care should not be implemented as a one-size-fits-all approach, but tailored to individual needs, including patients’ or relatives’ willingness to use it. Participants noted that the choice of the communication medium should be individualized: they recognized that some individuals might prefer telephone or text-based communication to videos. Technical reliability was considered essential, as any disruptions could distract from the encounter:

FG10P5: “I can’t hear you!” and so on. Half an hour goes by with: “Yes, try this, try that.” […] This isn’t the moment to be struggling with technical issues. […] This concerns a person’s life. The technical side should be secondary. It just needs to work.

For the same reason, participants stressed the need for healthcare professionals to be trained in the necessary technical skills. They further emphasized that, because tele-interactions strain human contact, healthcare professionals require strong communication skills to compensate, highlighting the need for targeted training. Participants also noted that the ICU environment can be emotionally intense for family members, and that offering follow-up support could be beneficial to help them process what they have witnessed:

FG3P1: I think we also need people to check in afterward, just to ask, “Is everything okay?” […]

FG3P5: Yes, yes. Seeing someone like that really hits you, I think.

FG3P1: Yeah… when my father was there, I took a photo. It’s somewhere on my phone, in some folder. Even now, I still can’t bring myself to look at it. You really have to be aware of what you’re seeing. That kind of support is so important.

They mentioned that telepalliative interactions may heighten this need, as family members are left in their own space after emotionally intense encounters, without healthcare professionals present to pick up on and respond to their distress. To help address this, they suggested that healthcare professionals should, when possible, encourage the involvement of other in-person support figures with family members, such as general practitioners, home care nurses, or informal support. Finally, participants emphasized that clear boundaries are needed to protect families’ well-being, with structured arrangements specifying when interactions occur:

FG3P5: Imagine being able to watch your sick grandmother 24 h a day. I’m just saying—that would be incredibly overwhelming and bring a huge sense of guilt. It makes you feel responsible, you know? Am I paying enough attention? […] Having someone in your family receiving palliative care is already really hard. And I think that’s why sometimes it’s actually better that contact is limited to just a key moment.

Similarly, participants highlighted that time limitations are important to safeguard ICU patients’ well-being, as they felt patients require sufficient rest due to their illness and may find it difficult to set boundaries with visitors.

### Theme 4: Practical ethical issues

Participants also highlighted practical ethical issues, including informed consent and privacy concerns, particularly regarding camera use. Finally, they also discussed equity and access issues, noting that although telehealth can possibly improve access to palliative care expertise in the ICU, certain barriers may still restrict who can use telepalliative care itself.

#### Sub-theme 4.1: Informed consent

If telepalliative care is used in the ICU, participants framed obtaining informed consent from the patient as an essential requirement (10/12 focus groups). Moreover, they stressed that informed consent should be dynamic, allowing patients to change their mind as they experience and come to understand telepalliative care in the ICU. Participants described a major difficulty in obtaining valid informed consent in the ICU, noting that patients are often unconscious. They explained that, although proxies are commonly used in other settings, this approach may be problematic in this context due to conflicts of interest, failing to reflect the patient’s true preferences:

FG3P1: When you’re emotionally involved, you almost always say yes […] But can you really make reasoned decisions and think, “Would they have actually wanted this?” No, you just want to see them—your husband, your daughter, your child—on screen.

Similarly, participants noted that advance directives would not be a feasible solution in this context, as anticipating the very specific circumstances of using telepalliative care in the ICU would be both difficult and overwhelming.

#### Sub-theme 4.2: Privacy

A central risk participants felt patients should be informed about is privacy (10/12 focus groups). Concerns focused particularly on the use of cameras and the feeling of being observed (10/12 focus groups). Participants highlighted that cameras could threaten personal privacy and disrupt intimacy, which are especially important during the final days of life. As one participant explained: “I think most people, if you asked around the world, would want to spend their last days in peace. Calm and serene, I’d say. Not with people peeking in.” (FG12P2) These concerns were reinforced by the possibility of recordings:

FG12P4: Yeah. Because nobody wants—sorry—to die on camera. I wouldn’t want that. […] Imagine they’d be recording their screen…

FG12P2: Ugh. (uneasy)

FG12P4: Yeah, no, I find that a really unsettling thought. […] I’d find it scary if my children—Inshallah—had a video, or an MP4 file, of me…

FG12P2: …in pain

FG12P4: …passing away.

Some participants felt that even the mere presence of cameras in patient rooms could infringe on privacy and limit intimate, spontaneous interactions between healthcare professionals and patients, or families and patients, due to uncertainty about when the camera is on. Participants further noted that cameras, if constantly running—for example, for remote monitoring—could create a strong sense of surveillance or control for patients. This concern was often summarized as “Big Brother is watching you.” (FG1P4, FG2P4, FG7P6). Participants also noted that this sense of surveillance could affect healthcare professionals if families observed care tasks virtually:

FG4P2: I was also thinking about the nurses’ privacy […] Imagine having to do your job while someone is constantly looking over your shoulder […] I don’t think I’d feel comfortable—not because I wouldn’t be doing my job properly, but just knowing someone is always watching or listening in on your conversations […]

FG4P4: It’s like you’re constantly taking an exam. Yeah.

To mitigate privacy concerns, participants proposed conditions for acceptable camera use. First, they emphasized that cameras should be used only for a clearly defined purpose and for limited periods of time. One suggested purpose was to support healthcare providers during care tasks:

FG7P6: If this is going to be used, I think that, from a privacy perspective, the healthcare provider in the room should be able to say, “Now I need support, now it can be on, and now it’s not needed.” It should be functional, not just running 24/7 for no reason.

Participants also suggested cameras could be activated at patients’ or families’ request to facilitate additional interactions with each other. Finally, participants raised concerns about the security of camera connections. One participant questioned whether these systems could adequately protect sensitive data. To address this, participants recommended: “It has to be done through secure apps. We’re dealing with sensitive information, after all.” (FG9P1)

#### Sub-theme 4.3: Equity and access

Participants emphasized the importance of ensuring a palliative care focus in the ICU. As one participant said: “Yeah, I think it’s totally natural that people in intensive care get so focused on saving lives that it’s easy to forget to take a step back and ask if going all-out is really the right move.” (FG5P1) Participants therefore pointed to the value of having access to palliative care expertise within the ICU: “Sometimes, bringing in someone from outside can make doctors take a step back and question how they—and their team—work, because otherwise, you just keep doing what you’ve always done.” (FG5P4) An ICU professional described such additional support as reassuring:

FG6P2: “Maybe the patient is restless […] and you’ve tried everything you usually do and still don’t know what to do next. It would be really helpful to just be able to reach out to someone who can go through it with you.”

Such support was described as particularly helpful in situations involving difficult end-of-life decisions, delivering bad news, or addressing complex psychological needs of the patient or family members. However, many participants suspected that ICU teams may not always have access to palliative care expertise. Across nearly all focus groups (11/12), participants mentioned telehealth as a promising solution when on-site consultation is not feasible: “In places like Canada or the U.S., where distances are huge, I think it could be really useful there.” (FG11P4) However, they stressed that on-site palliative care should be organized whenever reasonably feasible, and telehealth should not replace existing support. As one ICU professional explained:

FG7P7: Here in Belgium, where there’s practically a hospital in every backyard, I’d find it awful to have to call some center in Brussels for help. That really shows that the support I need isn’t here with me. How is that supposed to make me feel, standing at the patient’s bedside?

Still, participants saw intramural telepalliative care as useful when palliative care specialists are in other units, supplementing in-person ICU consultations. Similarly, this could be helpful to young doctors who might need to brainstorm with more experienced doctors, as one participant said “Something like an on-call telephone service” (FG4P1), but also to doctors who are alone on a shift and might need to consult with a specialist: “If a doctor is on their own and wants to consult with someone else, it could be a teleconsultation, like supervision or consultation with another doctor, or with nursing staff. I think we should move more in that direction, because it could really be valuable. […] You could share the relevant clinical parameters, explain the situation, and then make a joint decision together.” (FG3P4)

Although participants saw telehealth as a way to improve ICU professionals’ access to palliative care expertise, in many focus groups (10/12) potential barriers were discussed that could limit who can access telepalliative care itself, affecting patients, families, and staff. For example, participants in most focus groups noted that all users need an adequate level of digital literacy, with older generations at particular risk. One participant said: “My parents, for example, aren’t really familiar with this stuff, you know […] When I video-call my mom, I mostly just see her forehead and the ceiling.” (FG6P2) For this reason, participants emphasized that telehealth equipment must be highly usable, not only for patients and family members, but also for healthcare professionals often under high-pressure ICU conditions. Participants also mentioned other access barriers. Some were concerned that limited language proficiency could hinder use, because telehealth reduces nonverbal cues, such as gestures or facial expressions. For the same reason, functional limitations, such as hearing problems, were also a concern:

FG10P2: My mom doesn’t hear very well […] And when she has to talk to a screen, a lot of the communication just gets lost […] In real life, she can lip-read more easily […] She wouldn’t be able to use it.

Participants also highlighted that other functional limitations—including reduced strength, tremors, or vision impairments—could make using devices difficult, and that these are common in ICU-palliative care patients. Finally, participants noted that access to telepalliative care depends on having appropriate devices, potentially excluding patients, families, or hospitals with fewer resources. Participants were concerned that hospitals most in need of ICU telepalliative care, such as smaller or rural facilities, might be left without adequate support: “If you’re going to develop something like this, you’ve got to do it for the right people. But it’s never really for the right people, because that’s not where the money is.” (FG12P1)

## Discussion

This focus group study explored the ethical aspects of telepalliative care in the intensive care unit from the perspective of citizens. Citizens did not strongly oppose telepalliative care in the ICU, but they were cautious and emphasized the need for clear conditions: it should not replace in-person care as a standardized, one-size-fits-all solution, but instead be applied on a case-by-case basis that considers the specific care context. In other words, ethical acceptability is not determined by a general judgment of the technology itself, but by a particularized evaluation of its use in each specific situation. For example, citizens noted that the care setting matters, as telehealth is perceived as a more intuitive fit within the intensive care context than within palliative care. Closely linked to this, the interactional context is also important: citizens expressed full support for telepalliative care when interactions have an informational or task-oriented focus—such as exchanging medical information or coordinating practical arrangements—but were more skeptical about its use for relational, human-centered interactions. Still, citizens did not reject telehealth for relational, human-centered interactions altogether, noting that in exceptional situations—such as when in-person contact is not feasible—it may even support human contact.

These findings have both practical and theoretical implications for the ethical evaluation of telepalliative care in the ICU. From a practical perspective, citizens’ emphasis on the particular context suggests that ethical evaluation should not be confined to a one-time implementation decision, but should remain an ongoing, situation-specific process throughout its use. This approach resonates with a dynamic understanding of informed consent, in which consent is revisited and renegotiated over time as circumstances change, rather than treated as a single, one-time authorization — an approach that has also been advocated in the telepalliative care context [36–38]. This also implies that ethical acceptability may vary not only between patients, but also between different forms of telepalliative care for the same individual. For instance, a patient may accept telemonitoring while opposing teleconsultations with clinicians. Similarly, a family member may be comfortable receiving remote updates about the patient’s medical status, while considering remote discussions about treatment goals inappropriate. Still, citizens’ emphasis on context did not imply that ethical acceptability is arbitrary. Emerging from their reflections is a clear, practically applicable guiding question for case-by-case evaluation: does telehealth support humane care in the situation at hand? From a theoretical perspective, citizens’ emphasis on particular context aligns closely with a care ethics approach—an ethical perspective that prioritizes attentiveness to individual circumstances, supporting nuanced ethical evaluation [39]. Interestingly, the ethics of telepalliative care literature has largely relied on principlist frameworks [19], which emphasize general ethical principles—such as autonomy, beneficence, non-maleficence, and justice—often with less attention to particular circumstances [40]. We would therefore argue that a care ethics perspective might be an interesting lens for evaluating telepalliative care in the ICU.

A key finding is that citizens worry that human contact in tele-interactions may not achieve the same quality as in in-person encounters. They attribute this primarily to the absence of a physical dimension, mentioning a lack of physical presence, touch, sensory engagement, and non-verbal communication. Citizens also present this absence as ethically problematic, as it may undermine relational dimensions of care such as trust, respect, and the ability to provide comfort. This concern also aligns with care ethics literature, which emphasizes the embodied nature of caring relationships, including bodily engagement and situated responsiveness to others [41]. Clinical research further indicates that touch interventions benefit patients physically (e.g. reducing pain) and psychologically (e.g. lowering anxiety) [42]. Given the ethical and clinical importance of physical presence in care, it is noteworthy that emerging technologies increasingly separate what has traditionally been intertwined: physical contact and human presence. For example, care robots can provide some form of physical contact without human presence, while tele-interactions allow human presence without physical contact. Still, these technological developments should not be interpreted as inherently detrimental to relational care. A recent review, for instance, found that robotic touch can yield physical benefits comparable to human touch, although mental health benefits appear more limited [42]. A review of telepalliative care interventions for cancer patients also found that while telepalliative care proved beneficial for symptom management, it bore no mental health benefits [17]. Similarly, citizens in our study saw telehealth as a way to support human contact in well-defined circumstances, such as when in-person contact is not feasible or when it provides additional contact options. This suggests that human presence has the potential to be relationally meaningful even in the absence of physical presence, at least to some extent. Nevertheless, these emerging forms of contact remain insufficiently studied, making their relational impact difficult to anticipate. This warrants caution, particularly when implementing such technologies in highly vulnerable contexts such as ICU palliative care, where the relational dimension is central. In any case, citizens’ emphasis on in-person contact supports the careful integration of telehealth as a complement, in line with the “high-tech, high-touch” approach described in telepalliative care literature [37, 43-45].

A final consideration concerns the limitations inherent in exploring the ethics of telepalliative care in the ICU through citizens’ perspectives. While this study provides valuable insights into societal concerns about this emerging health technology, it may leave some important potential benefits of ICU telepalliative care underrecognized. One contributing factor appears to be that, despite an extensive introduction to the topic, citizens seemed to have a limited perception of telepalliative care. Even after clarifying that palliative care can be provided alongside curative treatments and is not confined to the terminal phase, participants predominantly associated it with end-of-life care. Similarly, regarding telehealth, despite a broad introduction, participants focused mainly on video communication, framing privacy concerns almost exclusively around cameras. Other modalities—such as telemonitoring, message-based communication, and telephone follow-up—received comparatively little attention. Participants also emphasized interactions with patients and families, while professional-to-professional exchanges were rarely mentioned. In sum, participants’ understanding of telepalliative care appeared somewhat limited, centering largely on end-of-life care delivered via video to patients and families. Participants’ limited understanding is likely not only a result of their inexperience with this particular setting, but it might have also been a result of an interview guide and educational material that were not sufficiently effective in further mitigating this difficulty despite our effort to provide multiple examples beyond video-communication. The initial framing exercise might have also contributed to a limited view of telepalliative care. While it might have provided valuable background, it might also have influenced how citizens perceive telepalliative care. Nevertheless, when telepalliative care was understood primarily in this way, attention naturally shifted toward relational, human-centered interactions, such as sensitive conversations and farewells—situations in which participants expressed the greatest hesitation about using telehealth. By contrast, non-emotional more task-focused interactions received far less attention, even though participants tended to view these applications more positively when they were mentioned, possibly reflecting that attention gravitated toward more challenging uses. This pattern suggests that the framing through which telepalliative care was imagined may have shaped the concerns that were raised, potentially leaving other, more beneficial applications less visible. In particular, uses such as an ICU clinician consulting a palliative care specialist via video, phone, or messaging—possibly informed by telemonitoring data—may have remained underexplored in participants’ reflections. A second benefit of telepalliative care in the ICU that may be overlooked from citizens’ perspectives is its potential to improve both cost-efficiency and patient care by expanding access to palliative services. Because citizens’ views are shaped primarily by values and expectations of care rather than system-level knowledge, they tend to focus on the risk that economic motives could compromise care quality. Yet evidence suggests a more nuanced picture: palliative care can reduce unwanted treatments and better align care with patient preferences, simultaneously lowering costs for health systems, patients, and families, while improving care quality [46]. Finally, reflections on equity and access were predominantly grounded in Western healthcare contexts, leaving global perspectives—such as tele-support and expertise exchange in low-resource settings—less visible.

### Strengths and limitations

A key strength of this study is the substantial qualitative dataset, comprising 12 in-person focus groups with 64 participants across four out of five provinces in Flanders. Purposive sampling was used to ensure a wide variety of perspectives on telepalliative care in the ICU, deliberately seeking diversity in prior experiences and worldviews. This approach successfully captured perspectives from some groups that are often under‑represented in research, such as participants identifying as Muslim [47]. It also included a mix of participants with and without prior experience of intensive care, palliative care, telehealth, and digital technologies, enabling the exploration of both lived and broader societal perspectives. However, we must acknowledge the relatively high proportion of highly educated participants as well as the relatively low participation of individuals from underrepresented groups, which might have affected the results. Theoretical sampling allowed for iterative refinement of participant selection in response to emerging themes. The in-person focus group format supported rich interaction and collective reflection, allowing participants to build on and challenge each other’s viewpoints. Although qualitative research does not aim for statistical generalizability, it can contribute to other forms of generalizability, including transferability, naturalistic generalizability, and analytic generalizability [48]. The concepts and considerations that emerged from this study may be transferable beyond the specific setting of telepalliative care in the ICU. For example, similar ethical considerations may arise in other high-tech healthcare environments, such as pediatric and neonatal ICUs, where palliative care plays an important role and telepalliative care may provide similar opportunities and challenges. Similarly, the perspectives of citizens in our study may also provide relevant insights into other healthcare contexts where telehealth, although not necessarily telepalliative care, might be implemented. For example, patients admitted to the hospital for long-term care might benefit from additional ways to remain in contact with their families, while similar ethical concerns may arise regarding the use of telehealth for sensitive communication and significant decision-making. These perspectives may also have naturalistic generalizability, as citizens may recognize similar concerns and considerations in their own experiences with increasingly digitalized healthcare. Finally, the conceptual framework developed in this study may have analytic generalizability beyond telepalliative care in the ICU, as it provides a conceptual understanding of ethical tensions that may also be relevant to other health and social care contexts in which telehealth technologies are implemented. Rigor was ensured through multiple complementary strategies. These included the use of QUAGOL to provide a structured analytic framework, concurrent data collection and analysis, investigator triangulation during the conceptual phase, and peer debriefing both immediately after focus groups and during team discussions. Additional strategies included external feedback from experts in intensive care and bioethics, reflexivity through memo writing and team discussions, and iterative refinement of the conceptual framework in response to emerging data.

Several limitations should be considered. As with all focus group research, participants may have been influenced by group dynamics or social desirability, which could have constrained the expression of dissenting or highly personal views, despite moderation strategies to encourage broad participation. Further, the high participation of individuals with professional or personal experience with palliative care might have influenced the group dynamics and themes that emerged. No repeat focus groups were conducted, and transcripts were not returned to participants for comment or correction, both of which could have provided opportunities for member checking and validating the findings. Repeat focus groups were not feasible because arranging a second session with multiple participants at the same time and place across different locations would have been logistically impractical. Returning transcripts for review was not possible, because although participants heard each other’s contributions during the sessions, sharing verbatim written records would have extended access beyond the immediate group setting and created a more durable record, thereby raising heightened concerns about confidentiality. Finally, the sample included a relatively high proportion of highly educated individuals (both university and non-university), which may limit the diversity of perspectives from less-educated groups. This overrepresentation is partly a consequence of deliberately including professionals and others with prior experience of intensive care, palliative care, telehealth, or digital technologies.

## Conclusions

Citizens’ perspectives suggest that telepalliative care in the ICU offers potential, but its implementation requires careful ethical evaluation both before and during use. To ensure that the human being remains at the center of care, special attention is needed to safeguard human contact. In addition, practical ethical considerations, including informed consent, privacy, and equitable access, must be addressed. The acceptability of telepalliative care should be determined on a case-by-case basis and reassessed over time, rather than treated as a one-time implementation decision. Ultimately, ethical implementation requires defining clear and specific use cases of telepalliative care in the ICU rather than treating “telehealth” as an abstract concept.

## Supplementary information

Supplementary Material 1: Additional file 1: Focusing exercise.

Supplementary Material 2: Additional file 2: Topic guide.

Supplementary Material 3: Additional file 3: Individual Conceptual Scheme – Focus Group 10.

## Data Availability

The datasets generated and/or analyzed during the current study are not publicly available due to the sensitive nature of the qualitative focus group data and to protect participant confidentiality.

## Footnotes

1 In this article, “family” is intended broadly to include anyone important in the patient’s life, including close friends.

## Abbreviations

ICU: Intensive Care Unit
COREQ: Consolidated Criteria for Reporting Qualitative Research
QUAGOL: Qualitative Analysis Guide of Leuven
GDPR: General Data Protection Regulation
