# Supplementary material for: Citizens’ perceptions of the ethics of telepalliative care in the intensive care unit: a focus group study
Source: BMC Med. 2026 Aug 18;24:648. doi: 10.1186/s12916-026-05137-8 (PMC13760820; doi:10.1186/s12916-026-05137-8)

KU LEUVEN

# Digitale palliatieve zorg. Een stap vooruit?

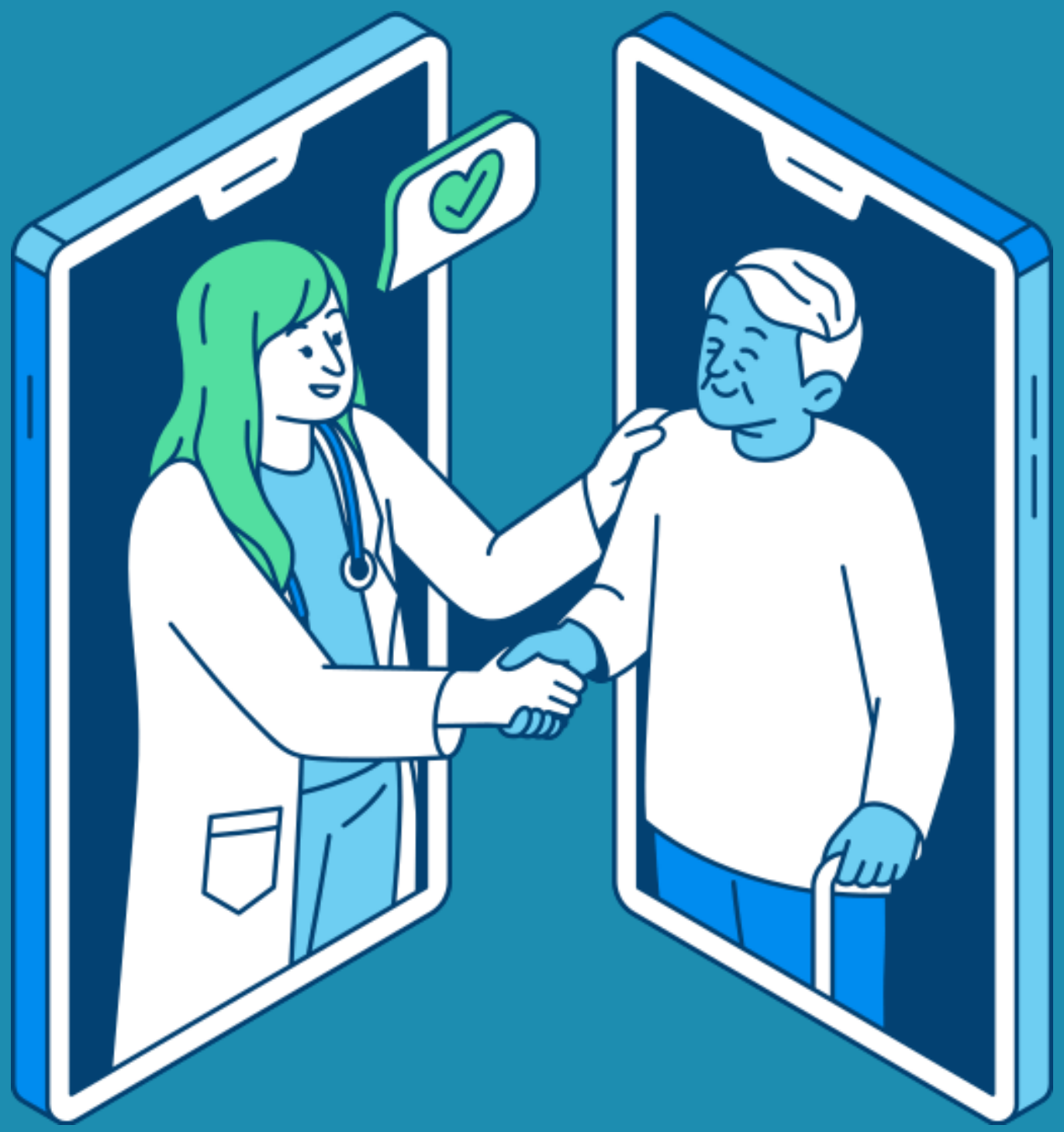

Gespreksleider: Leen Krieckemans

Observator: Jelena Vanhollebeke

Prof. Dr. Chris Gastmans
Prof. Dr. Michael Casaer
Dr. Alice Cavolo

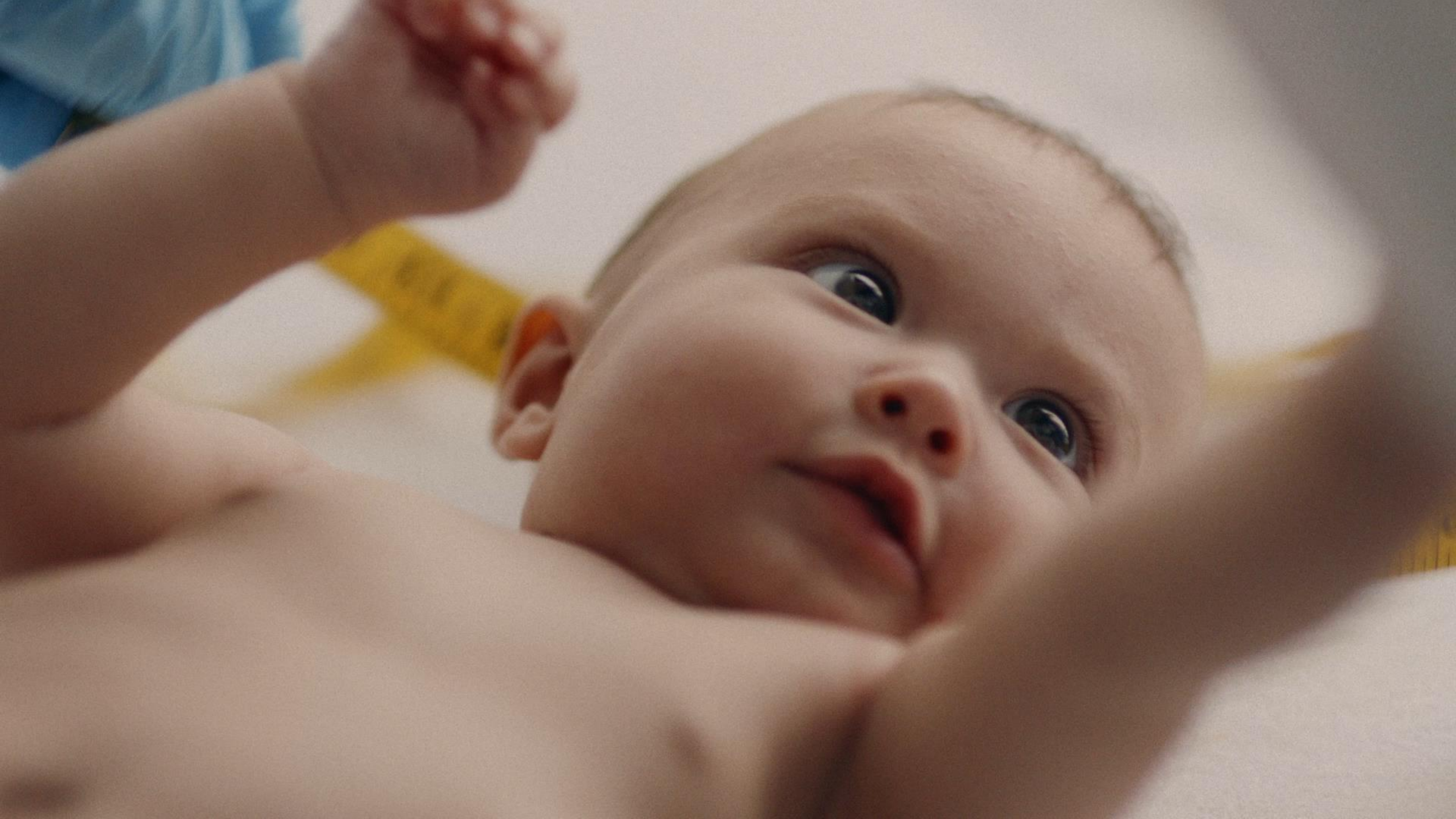

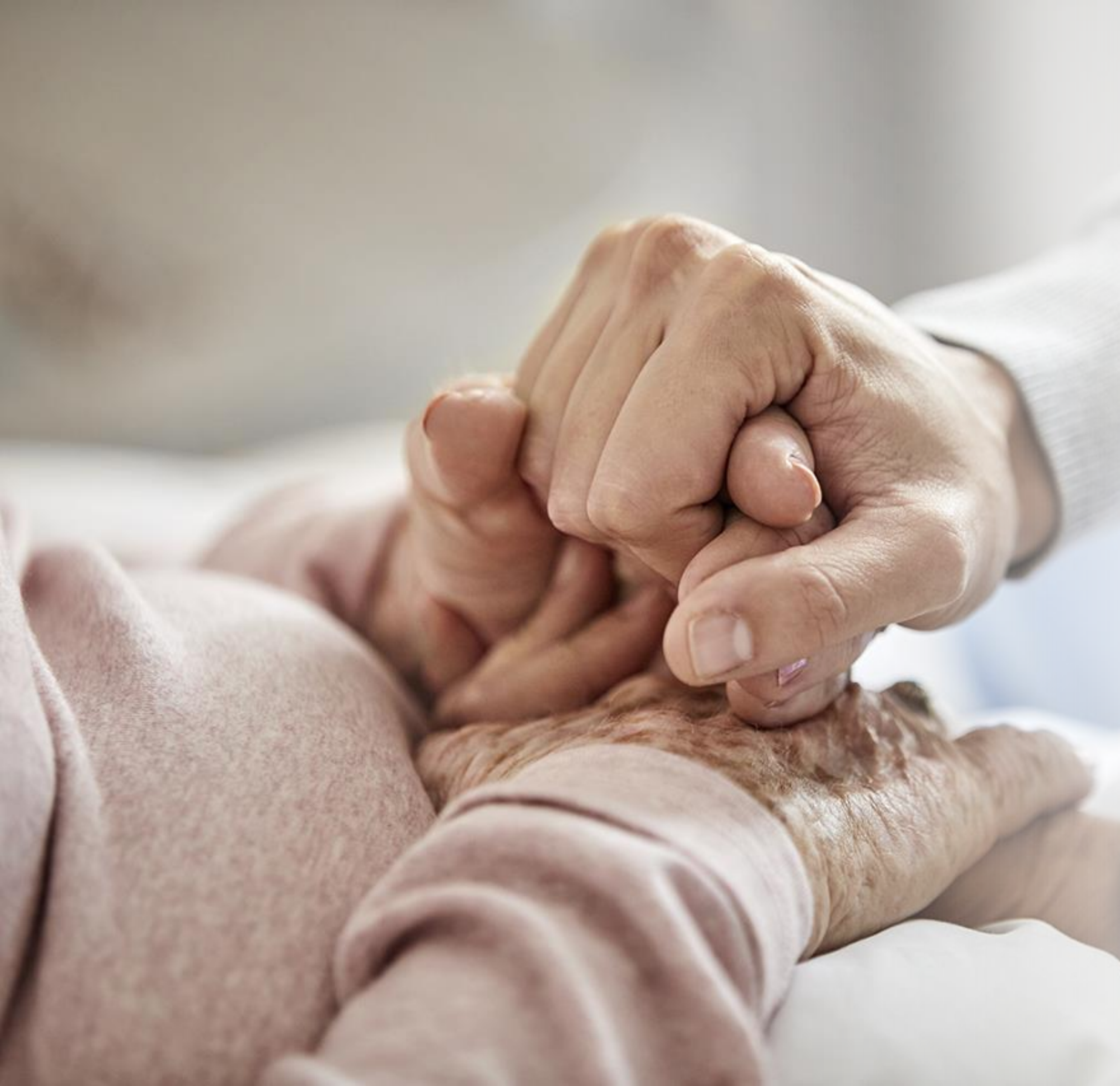

# Palliatieve zorg

Wie? Patiënten met een levensbedreigende aandoening en hun naasten

Doel: Zoveel mogelijk **levenskwaliteit en comfort**

# Palliatieve zorg is totaalzorg

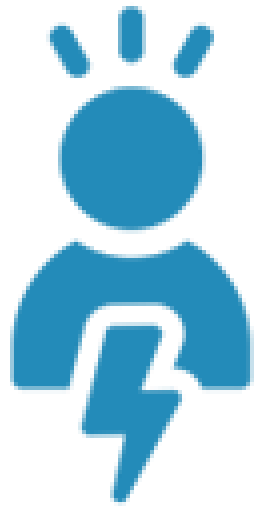

Fysiek

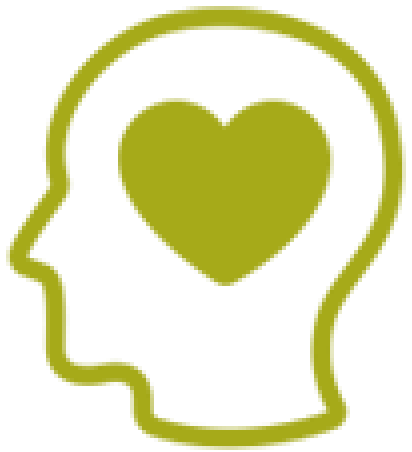

Psychisch

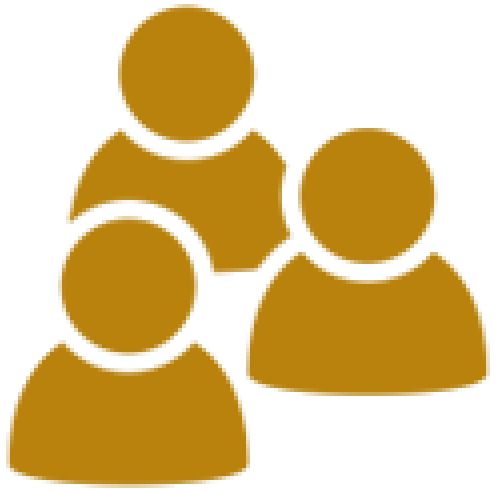

Sociaal

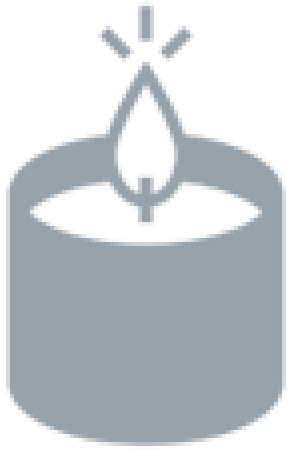

Spiritueel

De persoon staat centraal, niet de ziekte.

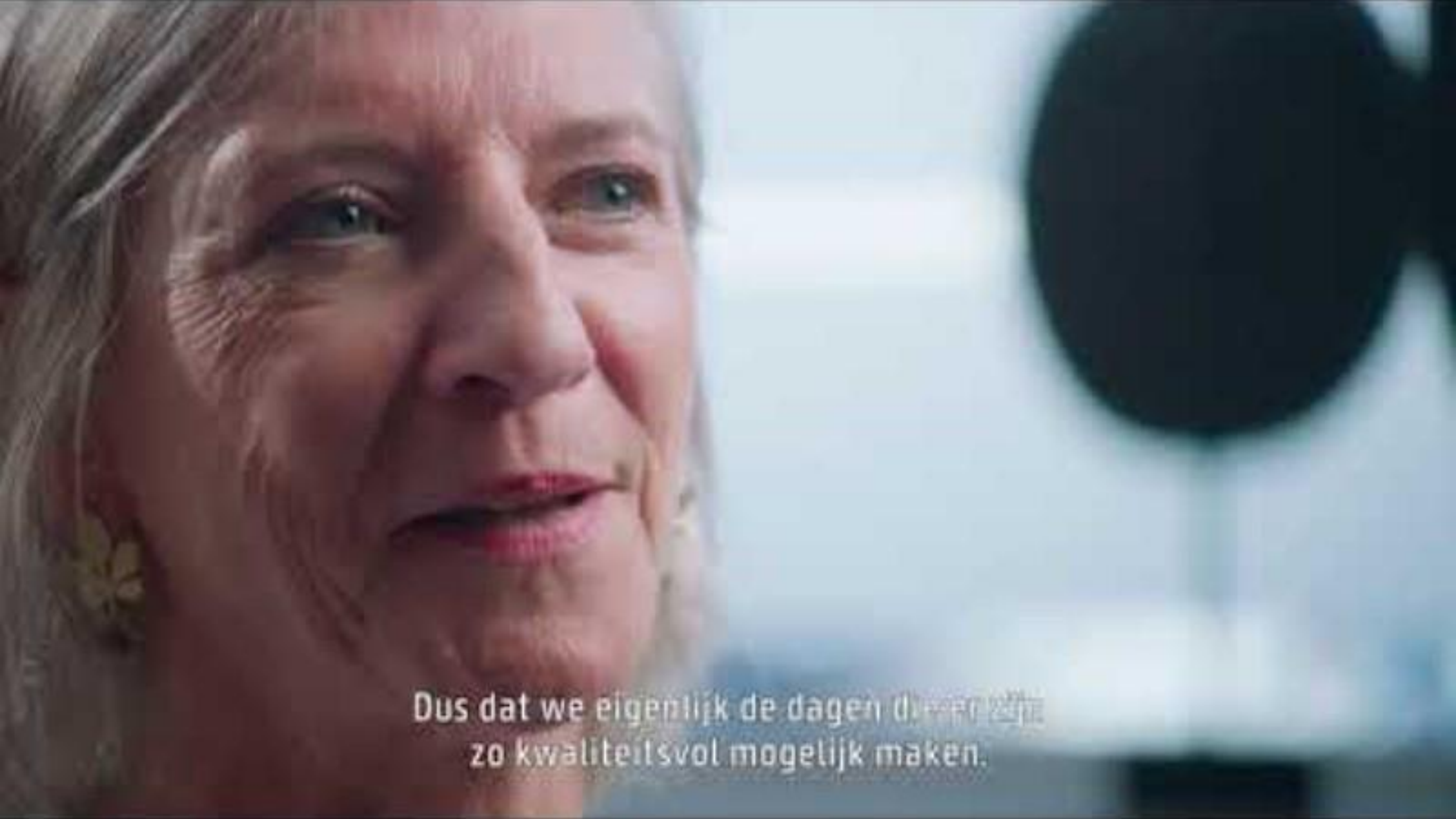
Dus dat we eigenlijk de dagen
zo kwaliteitsvol mogelijk maken.

# Voor iedereen, ongeacht…

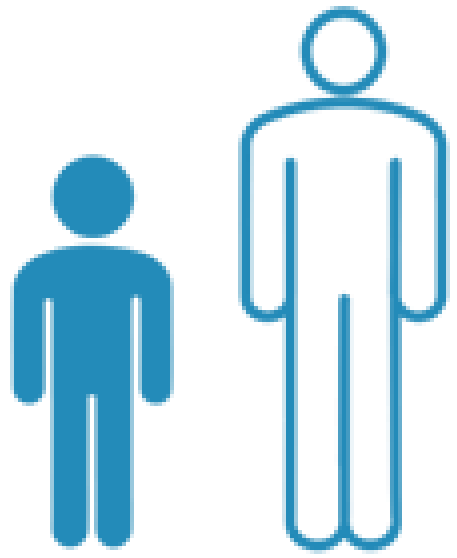

Leeftijd

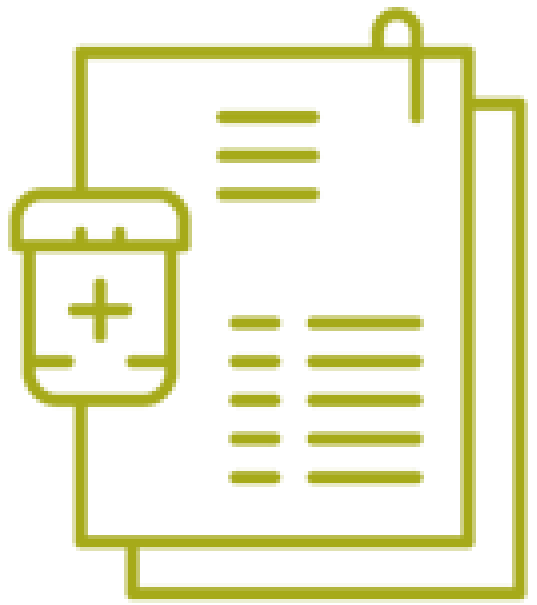

Ziekte

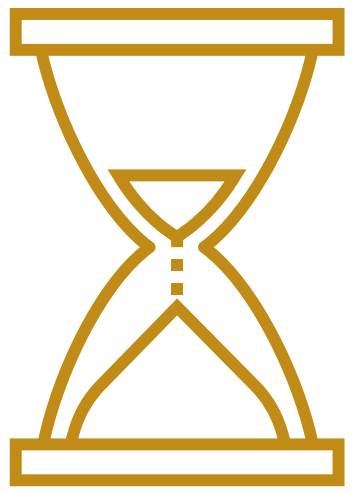

Levensverwachting

Palliatieve zorg kan hand in hand gaan met behandelingen die gericht zijn op herstel.

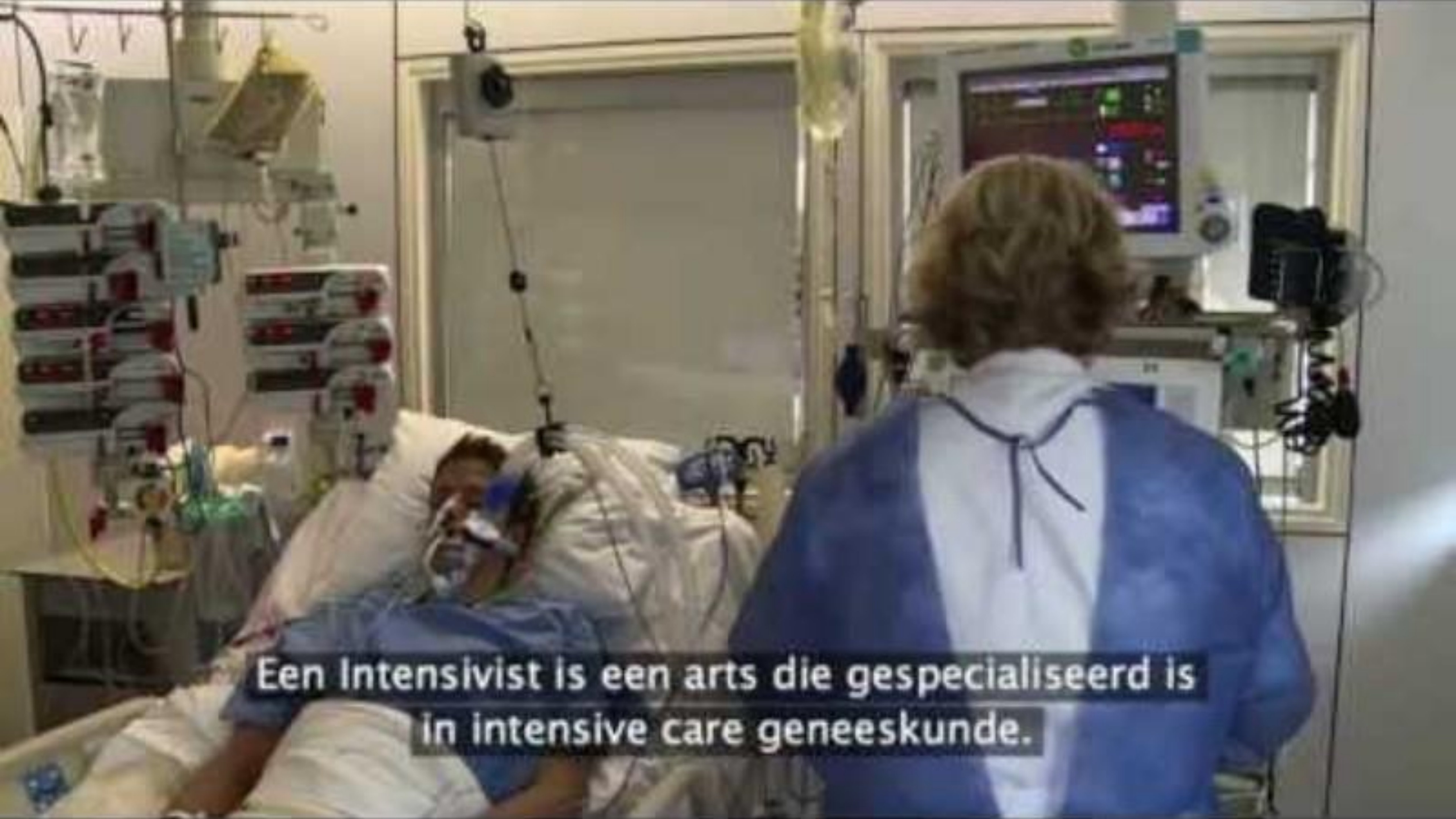
Een Intensivist is een arts die gespecialiseerd is
in intensive care geneeeskunde.

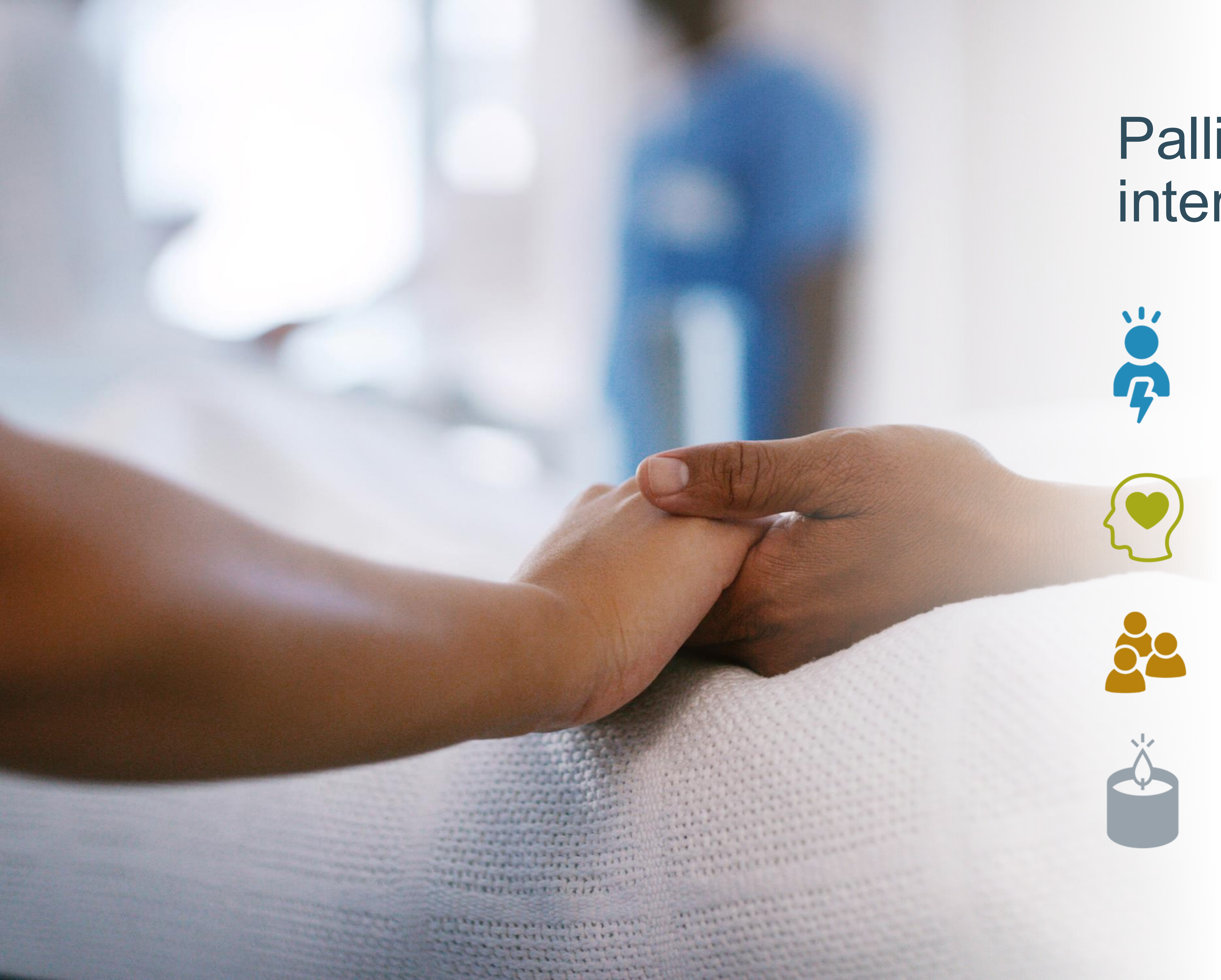

# Palliatieve zorg op intensieve zorg

- Symptoomcontrole (bv. pijn, benauwdheid, onrust, misselijkheid…)
- Psychologische ondersteuning voor patiënt en familie
- Zorgplanning
- Spirituele ondersteuning (bv. zingeving, rituelen…)

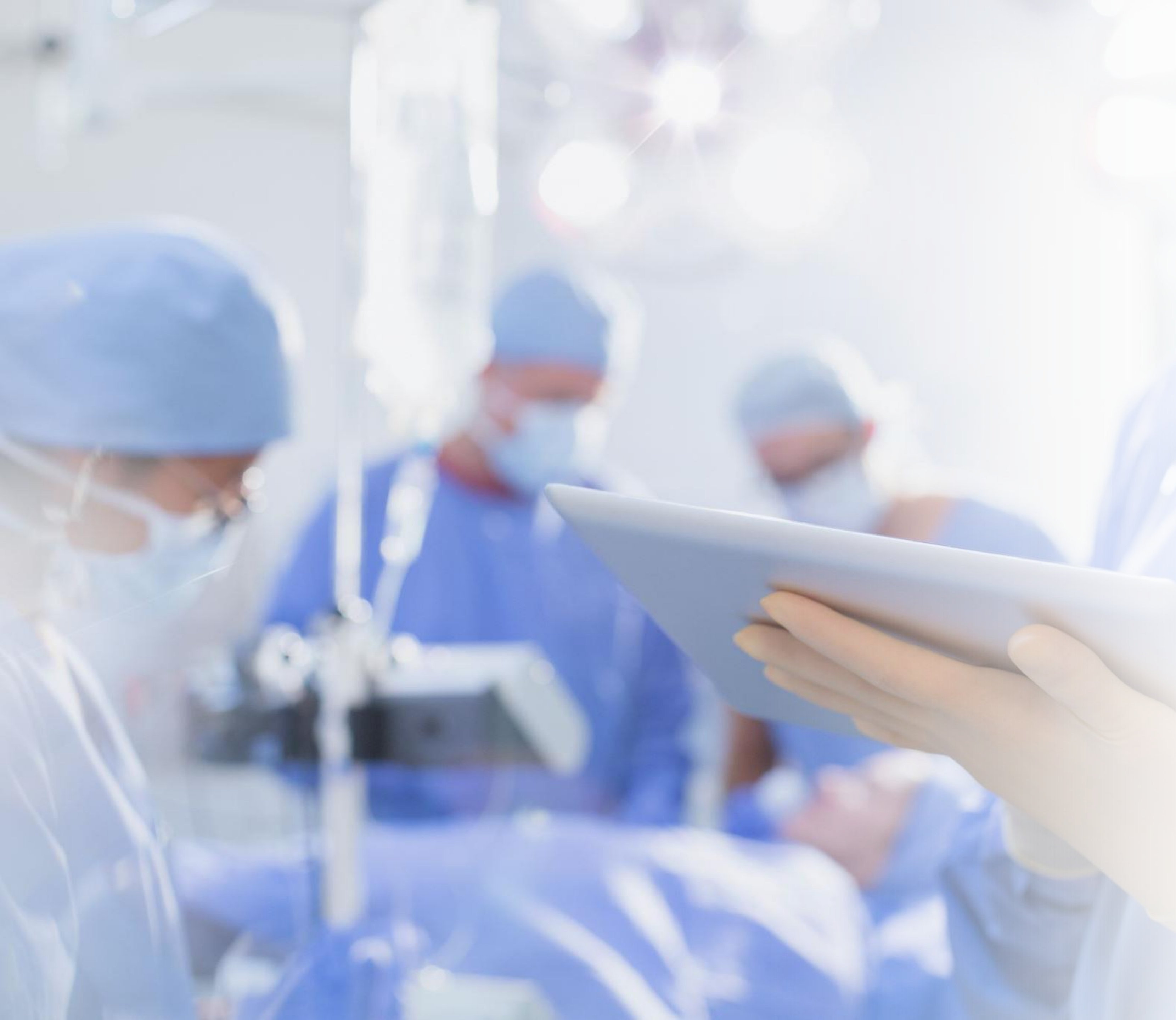

# Zorgplanning

Niet alles wat medisch mogelijk is, is ook gewenst.

- Open communicatie
- Samen beslissen

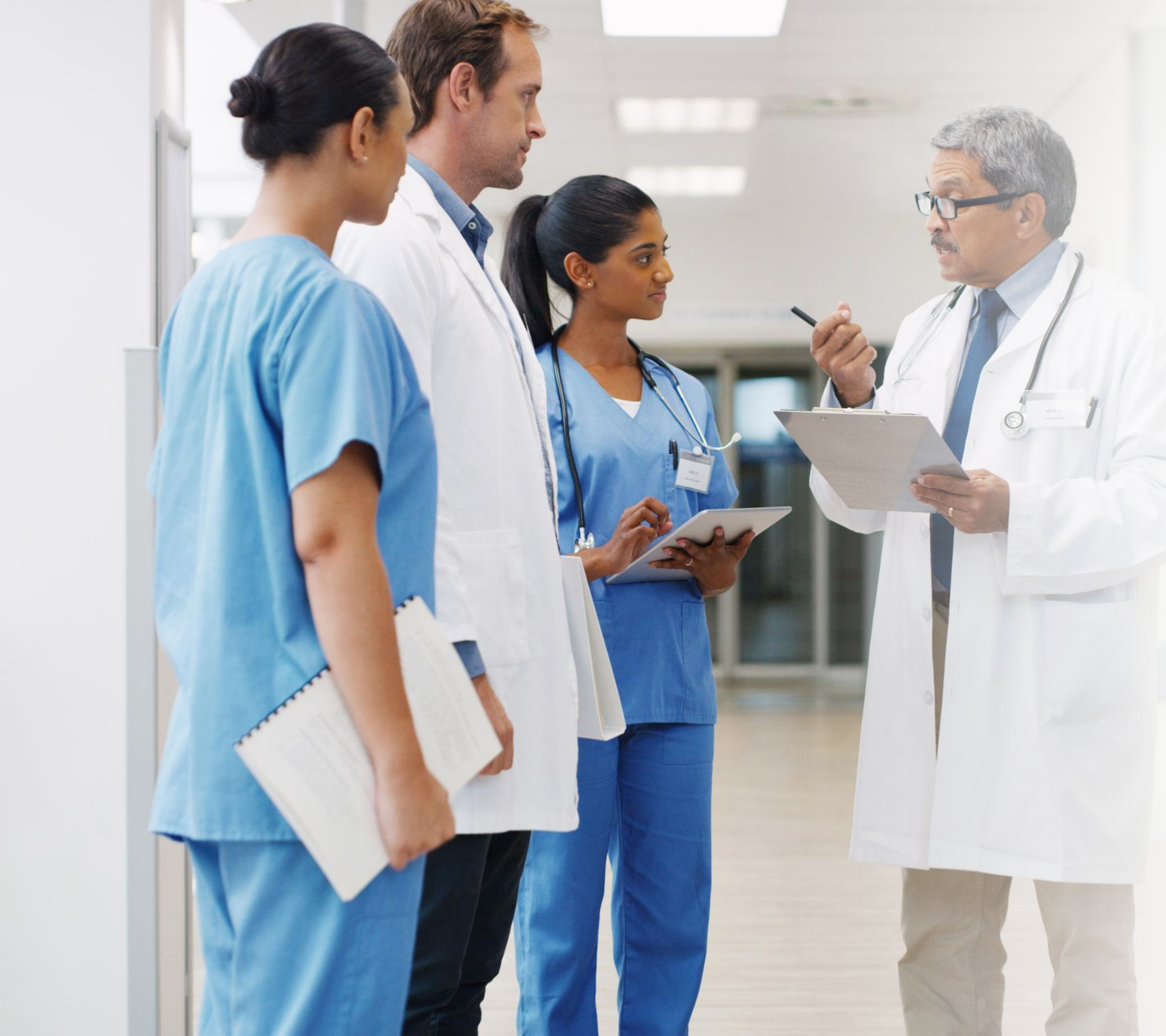

# Wie verleent palliatieve zorg op intensieve zorgen?

Het intensieve zorgteam zelf

Het palliatief supportteam

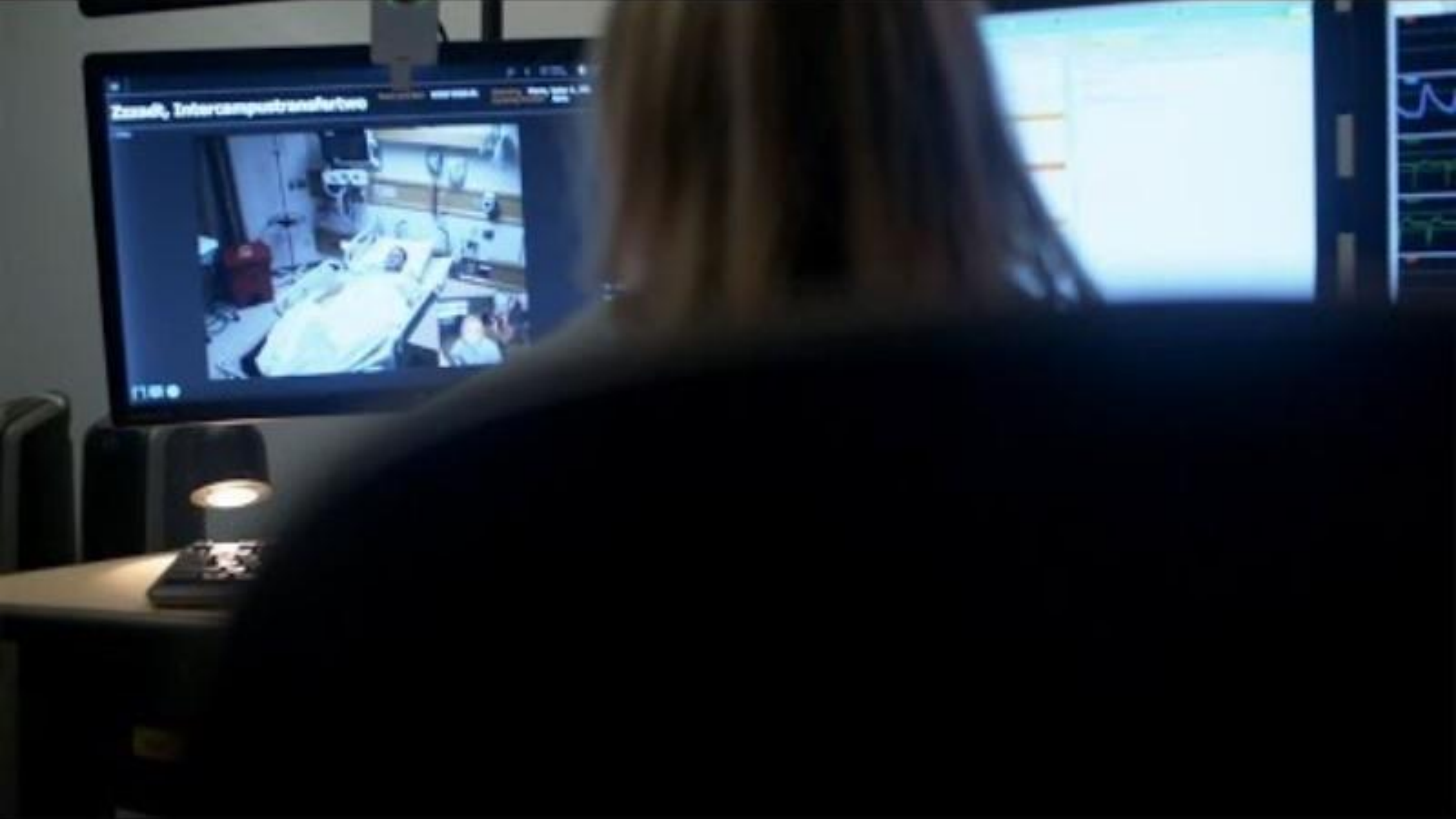
Intercampustransfertwo

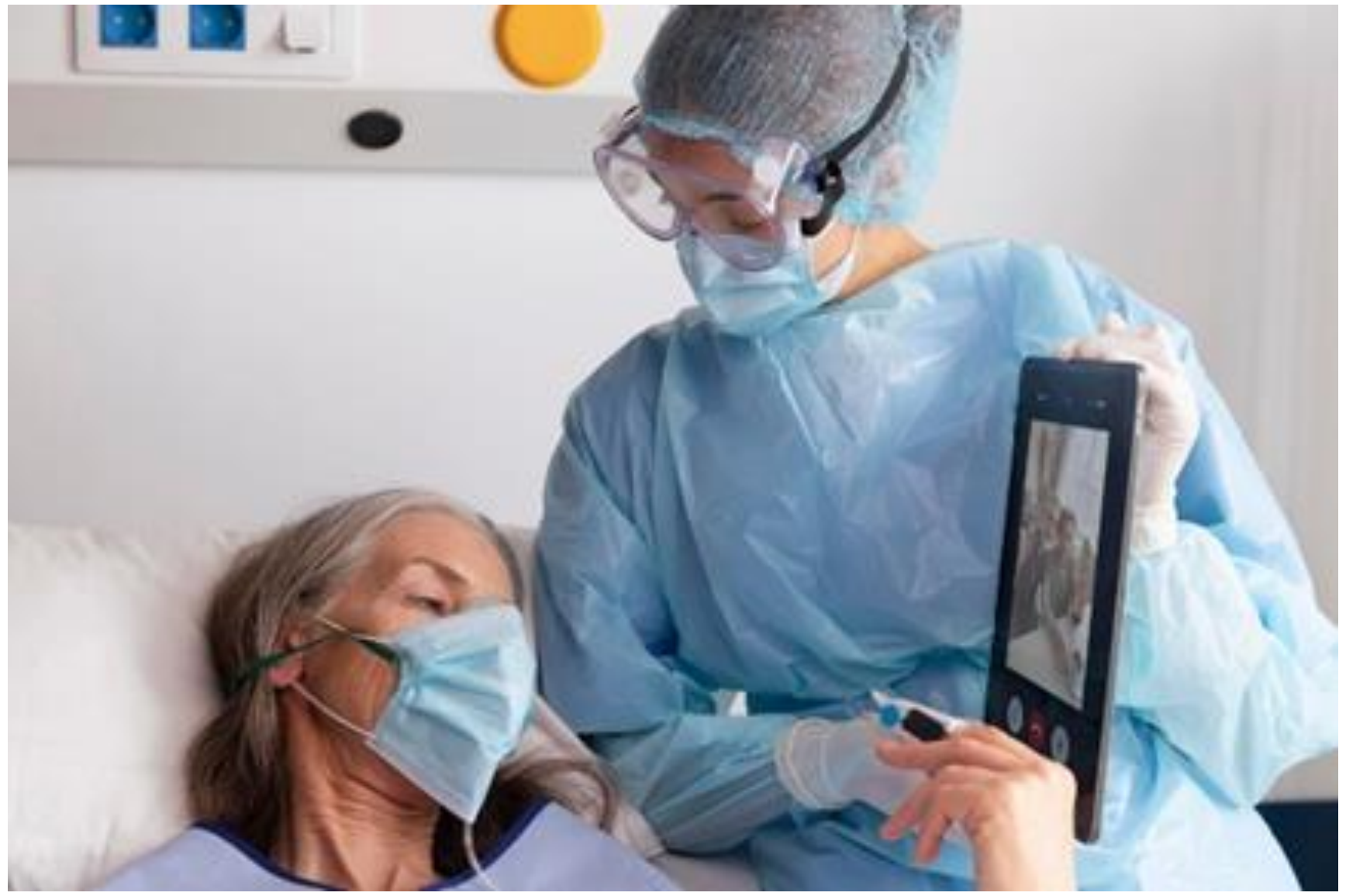

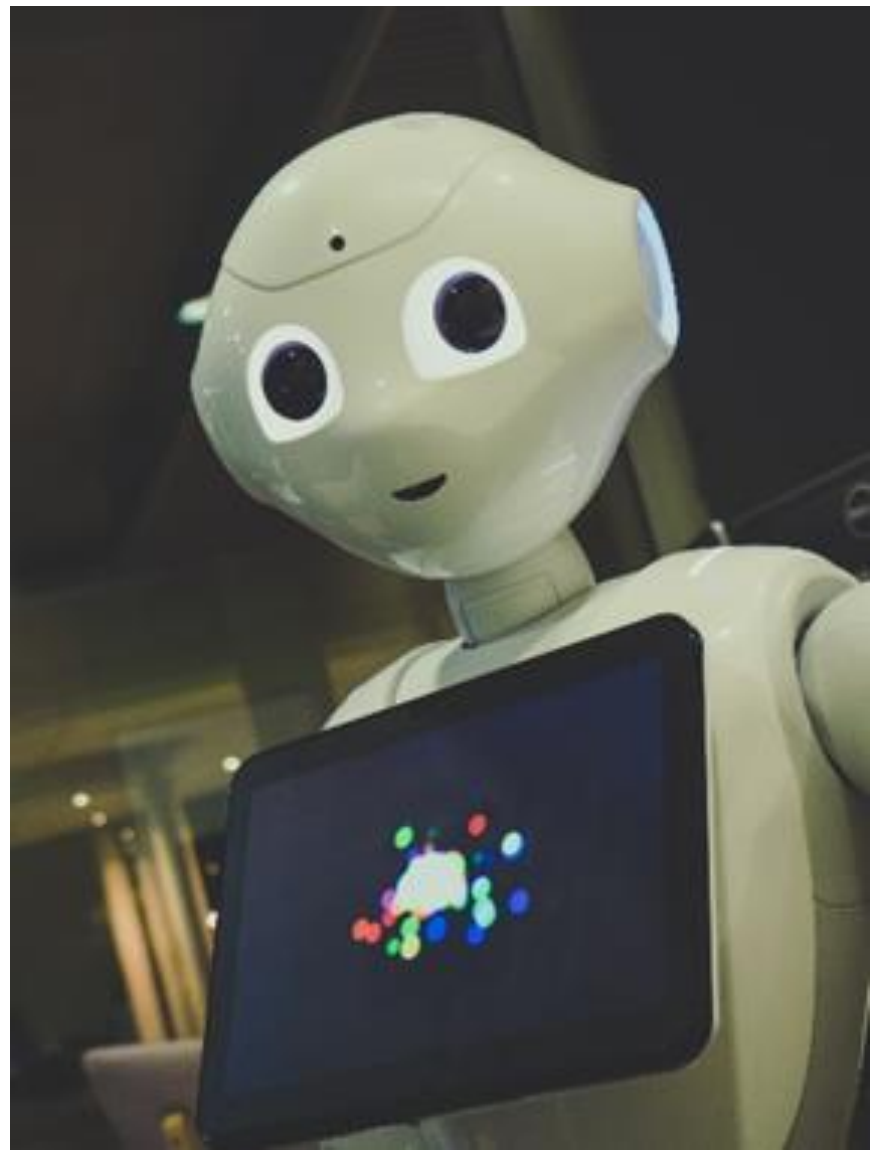

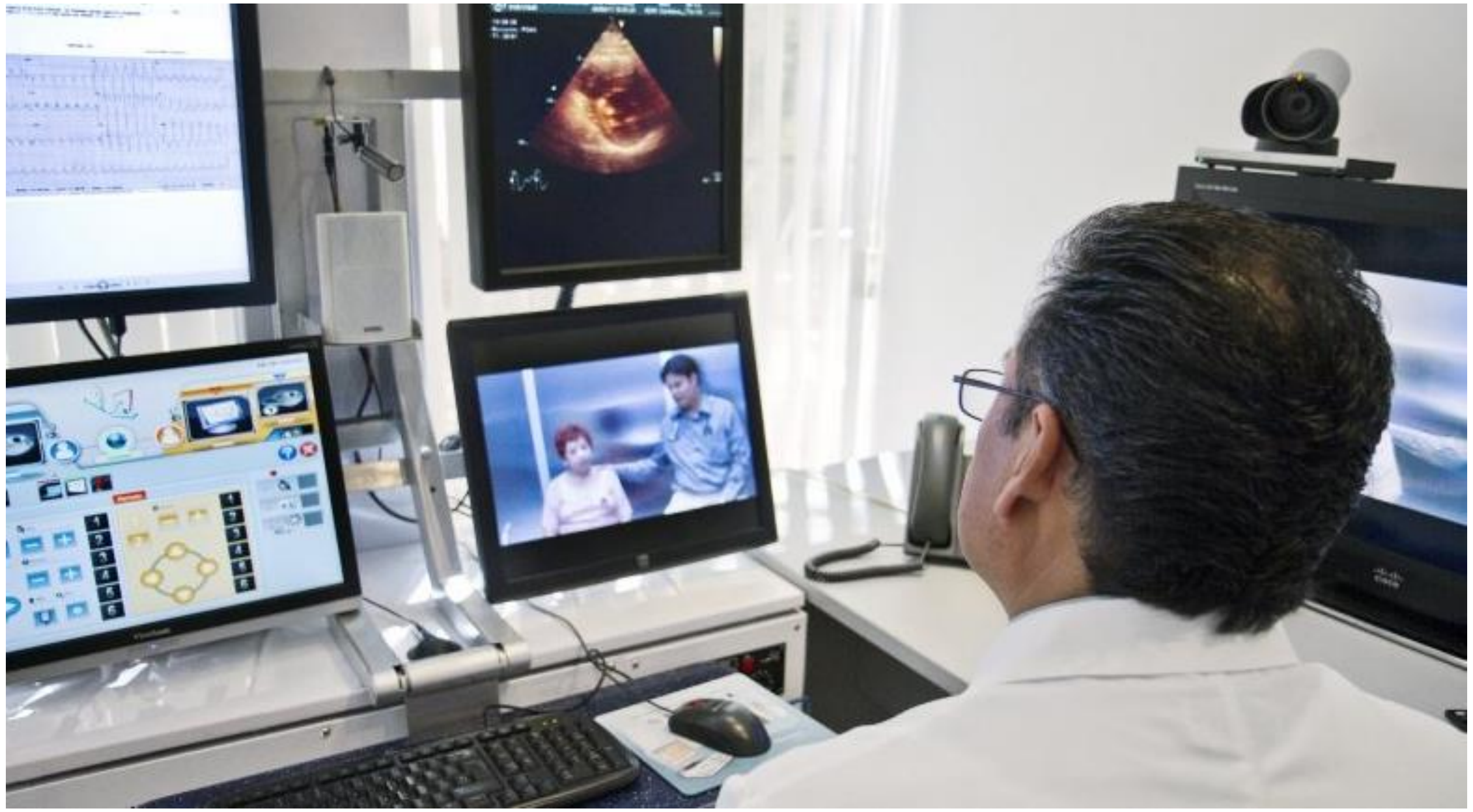

Supplement: Supplementary file 1 [file 12916_2026_5137_MOESM1_ESM.pdf]
