# Supplementary material for: Citizens’ perceptions of the ethics of telepalliative care in the intensive care unit: a focus group study
Source: BMC Med. 2026 Aug 18;24:648. doi: 10.1186/s12916-026-05137-8 (PMC13760820; doi:10.1186/s12916-026-05137-8)
Supplement: Supplementary file 2 [file 12916_2026_5137_MOESM2_ESM.docx]

**Topic Guide**

*English translation of the original Dutch topic guide.*

1. **Research question**

What do citizens perceive as the ethical aspects of telepalliative care in the intensive care unit?

2. **Introduction**

Welcome, and thank you very much for being here to participate in this focus group. We greatly appreciate your presence.

My name is Leen, and together with my colleague J., we will be facilitating this session. We are affiliated with KU Leuven, and this research focuses on the ethics of telepalliative care in intensive care. The input you provide today is very important for the study, and we value your time and willingness to share your thoughts.

Please feel free to share your thoughts and opinions. There are no right or wrong answers. Before we begin, we would like to ask for your consent to record the audio of this session. The recordings will be destroyed after transcription, and all data will be treated confidentially. We also want to remind you not to share any personal information about other participants outside of this session. Participation is voluntary, and you may withdraw at any time. The session will last approximately two hours, and there will be a short break.

The goal is to have a discussion about the ethics of telepalliative care in intensive care. Don’t worry if this topic is not immediately clear to you. We will introduce it step by step so you can gain a better understanding of what it involves. We will start with a few video clips and some background information. Feel free to ask questions or share your opinions during this phase as well.

This focus group is primarily about ethics and, therefore, about your opinions on this topic. However, it may touch on personal experiences and bring up difficult emotions. Please share only what you feel comfortable with, and know that it is okay not to respond to certain questions. I would also like to ask that you listen to each other respectfully.

Before we start, I think it would be nice to do a brief introduction round. You may say your name and briefly mention what interested you most about this topic.

3. **Focusing exercise**
4. **Main questions for focus group discussion**

General attitudes

Do you think telepalliative care in intensive care is **beneficial?** Why or why not?

What do you see as the **advantages** of telepalliative care? Which aspects might be easier to provide remotely?

What do you see as the **disadvantages** of telepalliative care? Which aspects might be more difficult to provide remotely?

**In what situations** do you think telepalliative care would be **appropriate**? When might it be inappropriate?

Are there **patients** for whom telepalliative care might be **more/less suitable**? If so, which patients?

Patient and family perspectives

If **a family member** (e.g., your spouse or parent) were in the ICU, would you feel comfortable with them receiving telepalliative care? Why or why not?

Imagine **you were in the ICU and needed palliative care**. Would you feel comfortable receiving it via telehealth? Why or why not?

How would you feel about interacting with a **healthcare professional** via telepalliative care while in the ICU?

How would you feel if a healthcare professional **monitored your vital signs** remotely?

How would you feel if a healthcare professional observed your condition via **camera?**

How would you feel if your ICU care team **consulted a palliative care expert via video,** for example to help manage your pain?

How would you feel about receiving palliative care from a **care robot**? How would you feel if a loved one received care from a palliative care robot?

How would you feel about discussing **advance care planning** via video, both as a patient and as a family member?

How would you feel about talking with a **psychologist** via video about emotional concerns (e.g., anxiety or sadness), both as a patient and as a family member?

How would you feel about discussing existential or spiritual concerns via video with a **chaplain or spiritual care worker**, both as a patient and as a family member?

How would you feel about your **family visiting** you via video while you are in the ICU? How would you feel about visiting a loved one in the ICU via video?

How would you feel about **saying goodbye** to your family via video at the end of life as a patient? How would you feel as a family member about saying goodbye to a loved one via video?

Would you prefer **telepalliative care or in-person palliative care**? Why? What would you prefer for a family member (parent or spouse), and why?

**What would help** you feel more comfortable with telepalliative care as an ICU patient?

**What could a telepalliative care provider** do to help you feel at ease?

Are there any **technologies** you feel more or less comfortable using?

Healthcare provider perspective

How would you feel about providing telepalliative care **as an ICU healthcare professional?**

How would you feel about providing telepalliative care **as a palliative care professional?**

Decision making

**Who should decide** whether telepalliative care is used in the ICU, and why?

Additional considerations

Are there any important topics related to telepalliative care in the ICU that we **haven’t discussed?**
