# Supplementary material for: Citizens’ perceptions of the ethics of telepalliative care in the intensive care unit: a focus group study
Source: BMC Med. 2026 Aug 18;24:648. doi: 10.1186/s12916-026-05137-8 (PMC13760820; doi:10.1186/s12916-026-05137-8)
Supplement: Supplementary file 3 [file 12916_2026_5137_MOESM3_ESM.docx]

**Ethical aspects of telepalliative care in ICU**

**Individual Conceptual Scheme – Focus Group 10**

Research question: What do citizens perceive as the ethical aspects of telepalliative care in the intensive care unit?

1. **Citizens perceive intuitive contradictions and parallels**

- Citizens perceive an **intuitive contradiction** between **intensive care** and **palliative care**:
  - They associate intensive care with **life-saving or life-prolonging** measures, while they associate palliative care with **respect for the approaching end of life**, emphasizing the quality of dying and saying goodbye.
  - They associate intensive care with a **cold and high-tech** environment, while they associate palliative care with **warmth and homeliness**.
    - However, there are also more and more initiatives to bring a sense of **homeliness into intensive care** , for example through the use of greenery
  - They associate intensive care with care focused primarily on the **physical,** while they associate palliative care with **holistic** care addressing physical, psychological, social, and spiritual dimensions.
  - They associate intensive care with a **fast pace of work and urgent/acute situations**, while palliative care for them is about **taking time and slowing down**.
- Citizens also perceive an **intuitive contradiction** between **telehealth** and **palliative care**

🡪 A combination of telehealth and intensive care could put pressure on the essential values of palliative care. In order to safeguard this core essence of palliative care, the focus group participants put a central criterion first.

2. **Telepalliative care in the ICU must always serve a well-considered purpose: The human being must be central**

- Telepalliative care in ICU should not be implemented blindly, but should always be (re)evaluated in the light of this central criterion
- People – patients and families **–** must be **at the heart** of telepalliative care in ICUs. The core of palliative care, namely **person-centred care**, must not be lost in the process.
  - Telepalliative care in ICU must be used where it represents **added value** for **patients or families**
    - Telepalliative care in ICU should contribute to better care or more care.
    - In the rollout of telepalliative care in ICUs, implementation should be prioritized in places where it offers the most added value for patients.
  - Telepalliative care should be used to promote **humanity**, and should **not dehumanize**.
    - Telepalliative care in ICU should promote care from people to people, without disrupting or replacing it
- Telepalliative care in ICU should **not have a purely economic purpose** .
  - Telepalliative care can provide greater **efficiency (in terms of resources, staff or time)**
    - **Any time saved** should be **dedicated to the patient.**
  - **Efficiency-driven thinking should not result in the patient being overlooked as a human being.**
- Telepalliative care in ICU should **not have a purely technological purpose** .
  - **Technology** should **not be an end in itself,** but **merely a tool** that must always be subordinate to the central goal, the human being.
  - Investments in technology must **always be weighed against investments in in-person care (such as training or recruitment, more staff, etc.)**
- 🡪 Telepalliative care in the ICU may be used to support **in-person palliative care,** but should **not replace** it as standard practice.

3. **Telepalliative care in ICU can promote patient autonomy:**

- Telepalliative care in ICU can promote patient empowerment
  - There is an opportunity to promote the autonomy of patients by giving them **more direct opportunities to initiate contact with family themselves** — for example, when a patient can independently start a video connection via an eye-controlled computer.
- Patients should have the choice of whether or not to opt for telepalliative care in ICU.
  - The patient must be able to decide for himself/herself whether telepalliative care in ICU represents added value for him/her.
  - This requires a case-by-case approach, not a one-size-fits-all.

4. **Telepalliative care in ICU can both promote and hinder human contact within palliative care**

- + Telepalliative care in ICU can promote human contact...
  - ... in emergency situations: by enabling some kind of contact **when physical presence is not possible or desirable** (e.g. due to distance, exhaustion related to commuting, hospital anxiety, covid...)
  - ... when it is used **in addition to in-person palliative care** (e.g. extra contact options outside visiting hours) and thus allows for more contact
- - Telepalliative care in ICU can hinder human contact within palliative care:
  - **Human contact** is **central** to **palliative care**. ("Being there" is often more important than "doing something")
  - How is this **disruption experienced in human contact** ?
    - By patient and family (as care recipients)
      - Experience of **distance** – Proximity under pressure
      - **Lack of comfort** and **reassurance**
      - Lack of **trust**
      - Lack of **humanity**
      - Lack of **warmth**; colder
  - Tele-contact does not constitute fully-fledged human contact
    - Lack of physical proximity (just "being there", often in silence)
    - Lack of touch and physical contact
    - Lack of emotion and empathy
  - Telepalliative care is less suitable for direct care, but may offer practical support

5. **Telepalliative care can both promote and hinder equitable access to palliative care in ICU**

- **Promote**:
  - Telepalliative care in ICU can promote access to palliative care by **bridging distance.**
    - For **professional caregivers**: when hospitals lack palliative expertise, telepalliative care in the ICU can still provide access to palliative expertise from another, more distant hospital.
    - For **family:** when family is not able to visit the patient in-person (e.g. because they are abroad, have mobility problems...), telepalliative care can still put family in touch with the patient.
    - For the **patient:** The patient, who cannot leave the ICU, can contact family himself.
  - ICU telepalliative care can promote access to palliative care by **providing** permanent availability
    - For **care providers:** ICU care providers have permanent access (also e.g. at night) to palliative expertise via telepalliative care in ICU
  - Technology offers opportunities to overcome language barriers, for example in the form of an **AI interpreter.**
- **Hindering:**
  - Telepalliative care in ICU can encourage **discrimination** because it is not equally accessible to everyone:
    - Discrimination based on limited **digital literacy**
      - The problem of digital literacy may gradually diminish as new generations become more familiar with digital technology.
      - But: technology **is evolving** so **fast** that it's hard to keep up
    - Discrimination based on **functional limitations**: critically ill patients often have hearing, vision, or mobility impairments, so telehealth technologies need to be adapted and user-friendly to ensure access
- The reimbursement for telepalliative care in ICU may be lower than for in-person palliative care, which may make healthcare providers less inclined to work within this framework.
